# A Systematic Review of Interventions Addressing Adherence to Anti-Diabetic Medications in Patients with Type 2 Diabetes—Impact on Adherence

**DOI:** 10.1371/journal.pone.0118296

**Published:** 2015-02-24

**Authors:** Sujata Sapkota, Jo-anne Brien, Jerry Greenfield, Parisa Aslani

**Affiliations:** 1 Faculty of Pharmacy, The University of Sydney, Sydney, NSW, Australia; 2 Department of Endocrinology, St. Vincent Hospital, Sydney, NSW, Australia; Karolinska Institutet, ITALY

## Abstract

**Background:**

The global prevalence of diabetes is increasing. Medications are a recommended strategy to control hyperglycaemia. However, patient adherence can be variable, impacting health outcomes. A range of interventions for patients with type 2 diabetes have focused on improving treatment adherence. This review evaluates the impact of these interventions on adherence to anti-diabetic medications and focuses on the methods and tools used to measure adherence.

**Method:**

Medline, Embase, CINAHL, IPA, PUBmed, and PsychINFO were searched for relevant articles published in 2000–2013, using appropriate search terms.

**Results:**

Fifty two studies addressing adherence to anti-diabetic medications in patients with type 2 diabetes met the inclusion criteria and were reviewed. Each study was assessed for research design, method(s) used for measuring medication adherence, and impact of intervention on medication adherence and glycaemic control. Fourteen studies were published in 2000–2009 and 38 in 2010–2013. Twenty two interventions led to improvements in adherence to anti-diabetic medications, while only nine improved both medication adherence and glycaemic control. A single strategy could not be identified which would be guaranteed to improve anti-diabetic medication adherence consistently. Nonetheless, most interventions were successful in influencing one or more of the outcomes assessed, indicating the usefulness of these interventions under certain circumstances. Self-report, particularly the Summary of Diabetes Self-Care Activities questionnaire was the most commonly used tool to assess medication adherence, although other self-report tools were used in more recent studies. Overall, there was a slight increase in the number of studies that employed multiple methods to assess medication adherence in studies conducted after 2008.

**Conclusion:**

The diversity of interventions and adherence measurements prevented a meta-analysis of the impact of interventions on adherence to therapy, highlighting the need for more consistency in methods in the area of adherence research. Whilst effective interventions were identified, it is not possible to conclude on an effective intervention that can be generalised to all patients with type 2 diabetes.

## Introduction

The global prevalence of diabetes is on the rise. Over 347 million people worldwide have been estimated to be suffering from diabetes [1]. The World Health Organization (WHO) projects that diabetes will be the 7^th^ leading cause of death in 2030 [2], and that the majority of the cases of diabetes will be type 2 [2]. Type 2 diabetes (T2D) is a chronic metabolic disease resulting from defects in insulin secretion and insulin action and glucagon suppression, which cause hyperglycaemia [3]. Adequate management of dysglycaemia in diabetes is extremely important, in particular to prevent or delay complications, and ensuring that patients have a good quality of life. Along with lifestyle management (such as diet and adequate physical activity) [4, 5], treatment with oral anti-diabetic agents and/ or insulin is recommended to control hyperglycaemia. Different classes of oral anti-diabetic medications are available for treatment, with metformin (a biguanide) being the optimal first line medication. Dual and then triple combination therapy is recommended if mono-therapy is insufficient. Due to progressive beta cell dysfunction, patients with T2D may ultimately require insulin replacement therapy to adequately manage their diabetes [5]. Therapy also needs to be individually tailored depending upon their age, co-morbidities and risks of developing complications [4].

Diabetes is further complicated by a multitude of other factors, such as, the ‘chronic’ nature of the disease, lifelong requirement for medications, requirement for changes in lifestyle, and the need to cope with social, cultural and psychological distress that may occur with the disease. In the midst of such complexities, remaining adherent to treatment recommendations may be a challenge [6]. Treatment adherence, in the context of diabetes, covers adherence to an array of self-care behaviours, constituting home glucose monitoring, adjustment of food intake, administration of medication(s), regular physical exercise, foot care and regular medical visits [7]. Although adherence to each self-care measure contributes to the effective management of diabetes, this review focuses only on adherence to anti-diabetic medications.

Adherence to oral hypoglycaemic medications in patients with T2D is 36 to 93% and to insulin is 63% [8]. The low level of medication adherence is likely to be one of the major factors contributing to sub-optimally controlled diabetes [9, 10]. Treatment non-adherence is well recognised, and interventions to promote adherence, improve glycaemic control, self-care behaviours and other key outcomes, have been designed and implemented. Several reviews and meta-analyses published over the last decade have addressed these interventions in patients with T2D [11–23]. Some reviews have focused on the wider aspect of treatment adherence in diabetes, incorporating adherence to anti-diabetic medications and treatment adherence as a whole [14, 19, 23]. Similarly, two reviews have discussed the interventions delivered by nurses [23] and pharmacists [14] to improve adherence to ‘medical treatment’. Where adherence to medications is specifically discussed [12, 16, 21, 22], one review included studies that analysed adherence to a range of medications taken by patients with T2D (anti-diabetic, anti-hypertensives and lipid lowering) rather than being specific to anti-diabetic medications [12]; two focused on analysing the methodological quality of the interventions [21, 22], and one focused on pharmacists’ interventions to improve adherence to oral anti-diabetic medications and included only controlled trials [16]. Thus reviews have to-date not focused on the range of interventions addressing anti-diabetic medication adherence. This review aimed to:

- investigate the impact of interventions on anti-diabetic medication adherence,
- identify measures of adherence used, and
- explore the changes in adherence measurement/ assessment methods used over time.

## Methods

### Literature search

A review of the literature was conducted to identify research articles that have evaluated the impact of interventions on adherence to anti-diabetic medications in patients with T2D. Studies were searched in the following databases: Medline, Embase, CINAHL, International Pharmaceutical Abstracts (IPA), PUBmed, and PsychINFO. Each database was searched using the appropriate terms for medication adherence (concept 1), type 2 diabetes/anti-diabetic medications (concept 2) and intervention studies (concept 3). Key words/terms were used to denote these concepts (S1 Fig.) and combined using ‘and’ operator (concept 1 and concept 2 and concept 3). The search strategy (S1 Appendix) was limited to English language articles published from January 2000 to April 2013 (inclusive). The references of relevant publications (all studies included in this review and relevant systematic reviews) [12, 14, 16, 19, 20, 22] were hand searched to find additional studies that met the inclusion criteria (S1 Table).

### Data extraction and analysis

For each study included in the review, the following ‘study characteristics’ were recorded: authors, year of publication, country where the study was conducted, study design, study population/sample, study site/setting, sample size, study duration, outcomes analysed and findings. Each study was analysed to determine whether medication adherence was the primary outcome measure, how medication adherence was measured, and adherence pre and post-intervention. The impact of the interventions on medication adherence and other outcomes were evaluated.

### Operational definitions

For the purposes of this review, the term ‘medication adherence/adherence’ was used and defined as the extent to which individuals take their medication. However, the terms used in S3 Table, S4 Table and S2 Appendix reflect those used in the reviewed studies. S2 Table demonstrates other operational definitions used in this review.

## Results

### 1. Study selection

The literature search identified 6,662 citations (S2 Fig.), of which 230 appeared to meet the inclusion criteria and were retrieved in full text. Of these, 49 met the study criteria and were reviewed. Three additional studies were identified from hand searching. Thus, a total of 52 studies [24–75] were included in the review.

### 2. Study characteristics

The studies included in the review were conducted in 15 different countries, with the majority (57.7%) conducted in the USA, followed by countries in Europe (17.3%), Asia (15.4%) and the rest of the world. Majority of the studies (n = 38) were published in the past 5 years (since 2009) whilst only 8 were published during 2000 to 2005. S3 Table outlines the characteristics of the studies presented in this review.

2.1. Study design and setting

Nearly half of the studies (n = 25) [25, 26, 28, 31, 32, 34, 36, 40, 44–47, 53, 56, 57, 59–61, 63, 64, 66, 69, 72–74] were 'randomized controlled' trials (S3 Table). However, a few studies utilised pre- and post-intervention designs [26, 34, 44]. Two studies were cluster randomized trials [42, 62], where either the participating general practices [62] or the clinicians [42] were randomized; four were controlled trials without randomization [27, 35, 48, 55], and one followed time-series design where the subjects served as their own controls [52]. Other studies varied in their designs and were cross-sectional [24, 41], quasi-experimental [33, 37, 43, 54, 75], and case series analyses [51]. The method of patient recruitment and research design were not clear in two studies [29, 70]. One study discussed the impact of two retrospective observational studies [65] and 8 studies were reported as pilot studies [24, 35, 37, 42, 47, 49, 61, 71].

Patients received the intervention mostly in community settings (n = 36) [24–27, 30, 33, 35–37, 40, 42–45, 47–50, 52–54, 57–59, 61, 64–72, 74, 75]. Other studies were conducted in hospitals (n = 6) [29, 32, 46, 51, 60, 73] clinics (n = 8) [28, 31, 34, 39, 55, 56, 62, 63], and one study involved multiple settings, community locations, clinic or patient’s home [38]. One study was not clear about the setting used [41].

2.2. Study subjects and recruitment criteria

Inclusion criteria varied between studies and included age, HbA1c level, taking an oral anti-diabetic medication and/or the duration the patient was on medication, and the duration since diagnosis with diabetes.

The subjects’ age groups varied and some studies were more inclusive, recruiting patients who were ≥18 years [31, 40, 48, 49, 53, 56, 73], or >18 years [35, 62, 72]. A vast majority did not specify the exact age [24–29, 33, 37–39, 41–43, 45, 46, 50–52, 58, 60, 63, 65, 69, 71, 75]. Others included adults who were ≥21 years [59], ≥30 years [64, 66], >40 years [30], ≥40 years [67], and ≥50 years [47]. A few provided age ranges for inclusion, such as, 18–65 years [44, 74], 18–70 years [36, 55], 18–75 years [34], 21–60 years [32], 21–80 years [68], 30–65 years [61], and 45–75 years [57]. One study included only the older adults 65 years and above [54], in contrast to another that included patients below 75 years of age [70].

The HbA1c level was sometimes specified as an inclusion criterion. However, the specifications varied and were >6.5% [44], ≥6.5% [50], >7% [26, 47, 58], ≥7% [55], ≥7.5% [36, 64, 69], 7–9.5% [42], 7–10% [61], ≥9% [31] and <8% [35].

Some studies used duration since diagnosis or taking an oral anti-diabetic medication as inclusion criteria. For example, patients with a diagnosis of T2D for at least 3 months [69], 6 months [38], one year [35, 42, 53, 68], 5 years [61], less than 10 years [32] prior to the study, or newly diagnosed (within 3–33 months of diagnosis) [45], were only included in the studies. One study only recruited 'newly diagnosed T2D patients’ without defining the duration since diagnosis [39]. Thirteen studies explicitly stated the requirement for the patients to be taking an oral anti-diabetic medication in order to be eligible for participation [29–32, 42, 47, 53, 57, 64, 66, 69, 72, 75].

A few studies focused on specific patient groups, for example: African American [24, 37, 47, 52, 71], Mexican American [34] and Hispanics/Latinos [38, 49]. Four studies [47, 51, 59, 66] included patients with diabetes and depression.

2.3. Sample size and study duration

Sample sizes were highly variable (S3 Table), ranging from 5 patients in a case-series pre/post analysis [51] to 5,123 patients in a prospective cohort pre/post analysis [67]. Likewise, the duration of the studies varied, from 10 weeks [49] to 4 years [43]. With the exception of one study [41], all other studies reported the duration of the intervention and/or the study. Most of the studies were conducted for 3 months (n = 14) [25, 26, 33–36, 46, 47, 50, 56, 60, 66, 68, 71], 6 months (n = 17) [24, 27, 29, 32, 37–40, 42, 44, 53, 57, 58, 67, 70, 74, 75], or 12 months (n = 10) [31, 45, 48, 52, 55, 59, 61, 63, 64, 72]. In one study [57], outcomes were also analysed eighteen months after the formal end of the study. In contrast, in a 6-month study, data were presented and analysed for a 3 month period only [75]. The maximum duration noted were for studies that involved the analysis of medical and pharmacy claims to determine the effect of ‘*value based plan design*’ [43]; the impact of U.S. Medicare part D [54], and the ‘*value based insurance design*’ programs [65] on adherence.

2.4. Outcomes analysed

A range of clinical and non-clinical outcomes were reported together with medication adherence, either as a primary or secondary outcome measure (S3 Table). With the exception of 14 studies [27, 29, 39, 41–43, 45, 54, 65, 67, 69, 70, 72, 74], all other studies analysed one or more clinical and/or biological outcomes. In addition, many studies also assessed patient specific outcomes, for example health related Quality of Life (QoL) [27, 59], diabetes specific QoL [32, 52] or QoL in general [60, 73]; diabetes knowledge [24, 32, 34, 38, 41, 42, 44, 49, 56, 68], and self-efficacy [34, 35, 37, 39, 40, 44, 45, 49, 56, 62, 68].

### 3. Assessing medication adherence

3.1. Medication adherence as a primary or secondary focus

In most studies (n = 38) medication adherence was assessed as a primary outcome [24–30, 32–35, 37, 39–47, 50–54, 56, 58, 61, 65–69, 71–74] (S4 Table). Adherence was measured as a self-management/ self-care behavioural outcome at the same time as other measures, for example adherence to diet, exercise, foot care, and blood glucose testing. Only 15 studies [25, 28, 41–43, 46, 47, 54, 60, 65–67, 69, 72, 74] assessed the impact of the intervention on medication adherence alone. Other studies specified medication adherence as a secondary outcome. Clinical measures (primarily the HbA1c) were the primary outcome measure in most of these studies [31, 48, 49, 55, 57, 59, 62–64].

3.2. Measuring medication adherence

Overall, the studies included measured adherence to medications as the measure of medication taking compared to the prescribed regimen (S4 Table). Some studies used ‘compliance’ to describe this medication taking. Only one study measured persistence to therapy in addition to adherence [42]. Studies reported adherence as number or proportion of adherent/non-adherent patients, adherence scores, or number of days the patient reported adherent behaviour.

Patient self-report, pharmacy refills and claims data and electronic measures were used to assess adherence. Six studies [28, 47, 51, 66, 69, 74] used electronic measures to assess adherence. Medication Event Monitoring System (MEMS) was used in five and the Real Time Medication Monitoring (RTMM) system in another [74]. Three of the six also used self-report [28, 51, 69]. Ten studies used prescription refill claims (pharmacy and medical claims data) [29, 30, 42, 43, 54, 57, 64, 65, 67, 72], with four also measuring adherence through self-report [30, 42, 57, 64]. Self-report alone was used in all other remaining studies.

Self-report was used in 82.7% of the studies (n = 43). Most (n = 16) [25, 27, 32–37, 44, 45, 49, 51, 52, 56, 64, 68] used the medication subscale (1 or 2 items) questions in the Summary of Diabetes Self-Care Activities (SDSCA) questionnaire [76] to assess adherence. A study [42] used ‘the number of days patient missed taking the pill in the last 7 days’ and another analysed the response to the question ‘have you missed a medication dose in the past week?’ [53], as opposed to the approach by SDSCA, which takes into consideration the number of days the patient was adherent to their medication in the last 7 days. Morisky Medication Adherence Scale (MMAS) [38, 53, 56, 59, 60, 64, 73], Medication Adherence Report Scale (MARS) [39, 45, 62, 69, 70], Morisky-Green test [48, 55], Brief Medication Questionnaire (BMQ) [46, 58] and Hill Bone compliance questionnaire [40] were other self-report tools used. A few studies reported the use of validated questionnaires without giving the details of the questions asked [26, 41, 61, 63, 75]. One study simply tabulated medication adherence using a ‘yes’ and ‘no’ response [24].

Medication adherence was measured as a single entity for the entire therapeutic class (anti-diabetic medications), rather than for individual anti-diabetic medication in almost all of the studies, except for one study that specifically addressed adherence to metformin [28].

3.3. Longitudinal changes in methods of assessing adherence

The self-report tools used to measure adherence have changed over time. SDSCA was used in all studies published during 2006 to 2008, compared to only 25% of the studies in 2000–2005. Since 2009, a range of approaches have been used to measure adherence. Although SDSCA has remained an important tool, other methods, such as the Morisky scales, MARS, Hill Bone compliance questionnaire, and BMQ have also been employed. Of 38 studies published since 2009, eight used SDSCA [44, 45, 49, 51, 52, 56, 64, 68], four [45, 51, 56, 64] of which also utilised an additional method to assess adherence, either another self-report tool [45, 56, 64] or an electronic measure [51]. While only two studies [28, 30], both published in 2004 used multiple methods to analyse adherence, seven studies [42, 45, 51, 56, 57, 64, 69], conducted (after 2008) used either two different tools or different methods for assessing adherence. Electronic methods, pharmacy claims and pharmacy refills have been reported.

### 4. Interventions and their impact

4.1. Impact on anti-diabetic medication adherence

S2 Appendix provides a description of the interventions and their impact. Forty nine articles dealt with interventions directed at the patient [24–26, 28, 30–48, 50–75], two directed at the healthcare provider [27, 29], and one directed at both [49]. Twenty two studies [28, 29, 33, 41, 46–51, 53, 54, 58, 60, 64, 66, 67, 69, 71–74] reported improvements in medication adherence. In two of these studies [28, 69] there were contradictory results between the two methods of measuring adherence. Rosen et al [28] reported a significant improvement in medication adherence when MEMS was used, but the improvement was not significant when assessed using ‘self-report’. Furthermore an improvement was detected using MEMS at the 16^th^ week i.e. immediately after the intervention, and a decline in medication adherence was observed in subsequent analysis after 16 weeks. Similarly, Farmer et al [69] demonstrated that medication adherence improved significantly when analysed objectively (using MEMS) and but did not when self-report (MARS) was used.

Two studies [46, 48] reported a significantly positive impact of their interventions on adherence, however, did not include their control groups in the analysis. Therefore, they did not report how the interventions impacted adherence when taking into account the control groups’ data. A study on the impact of SMS showed an improvement in the number of doses taken within an agreed time period for the intervention group in comparison to control [74]. However, the intervention did not have a significant effect on days without dosing and missed doses.

In addition, a few comparable intervention strategies delivered different results; for example, continuous education and reinforcement text messages delivered to patients based on their blood glucose level, significantly improved medication adherence [33]; however, a similar intervention consisting of tailored feedback and reminders based on patient-specific data via messages on cellular phone failed to show an impact on adherence [35]. In both studies nurses delivered the interventions. Likewise, while a regular multidisciplinary education program positively influenced medication adherence in Nigerian patients [41], two educational sessions on diabetes self-management failed to impact medication adherence in Korean immigrants in the USA [68]. Furthermore, pharmacist delivered ‘care plans’ have been effective [48] in one study and ineffective in another [30].

4.2. Impact on clinical outcomes

HbA1c was an outcome measure (S3 Table) in 34 studies [24–26, 28, 30–38, 40, 44, 47–53, 55–57, 59, 61–64, 66, 68, 71, 75]. Nine studies reported a positive impact of the intervention on both medication adherence and HbA1c [33, 47–51, 53, 64, 66]. Sixteen of the 34 studies [30, 33, 36, 47–52, 55, 57, 61, 63, 64, 66, 68] reported a significant impact of intervention on HbA1c levels. In two studies however, the significant impact was observed only for part of the duration of the intervention [55, 63]. Garcia-Huidobro et al [55] demonstrated a significant positive impact on HbA1c during the 2^nd^ 6 months of the 12 month intervention period. Wakefield et al showed a significant impact in the first 6 months which was not sustained at 12 months [63].

A small number of studies reported significant improvements in HbA1C levels of participants in the intervention groups, however, the difference was not significant when control group data were taken into consideration (between group differences were not statistically significant) [26, 38, 53]. Furthermore, in one study [53] the improvements observed in HbA1c levels were only significant amongst the patients who had baseline HbA1c ≥7.0 and not for the entire sample.

Other clinical or biological parameters measured varied between the studies and included blood glucose levels, blood pressure, and lipid profiles (S3 Table). Twenty five studies measured one or more clinical/biological parameters other than HbA1c [25, 30, 34, 35, 38, 40, 44, 46–50, 55–63, 66, 68, 71, 73], of which eighteen reported improvements in one or more parameters [30, 34, 38, 44, 47–50, 55, 58–61, 63, 66, 68, 71, 73]. In three [51, 59, 66] of the four studies [47, 51, 59, 66] that included patients with depression and diabetes, the interventions were successful in reducing depression symptoms. Blood glucose was improved in six studies [34, 48, 50, 58, 60, 73].

4.3. Impact on non-clinical outcomes

A range of non-clinical outcomes were evaluated highlighting the diversity of interventions and the range of non-clinical outcomes important in diabetes (S3 Table). A majority (94.2%) of the studies reported an impact on one or more non-clinical outcomes. Improvements were observed in adherence to exercise [25, 33, 44, 49–51, 53, 57], diet [26, 39, 44, 50, 51, 57, 71] blood glucose testing [26, 49, 51], foot care [33, 44, 49, 51, 57, 71], QoL [27, 73], self-efficacy [34, 35, 44, 45], patient knowledge [30, 32, 34, 41, 42, 49, 57, 58, 60] and goal achievements [37, 44].

Other medication related issues were influenced by some interventions, such as, identification and resolution of medication discrepancies [25, 36], improvements in medication appropriateness index [31], identification of drug therapy problems [48], pharmacotherapeutic changes [57], and intensification of oral diabetic therapy [56].

## Discussion

A number of studies were identified which had investigated the impact of interventions on adherence to anti-diabetic medications, as well as a range of clinical and non-clinical patient outcomes. Only a few interventions demonstrated a significant positive impact on adherence to anti-diabetic medication, and even fewer had shown improvements in both medication adherence and HbA1c levels. A range of tools were used in the studies to evaluate adherence, and consequently adherence was reported in several ways (e.g. proportion of adherent patients, adherence scores), preventing meta-analyses or direct comparisons of the findings. Despite the variability in study designs observed, including subject inclusion criteria, a range of sample sizes and variability in the duration of time since diagnosis, the review has highlighted an overall improvement in medication adherence and other secondary outcomes as a result of some interventions delivered to patients. Interestingly, the review also revealed a change in the tools and methods used to evaluate adherence to therapy, in particular, over the past 5 years.

### Study characteristics and methodological limitations

There has been a significant increase in the number of studies evaluating the impact of interventions on anti-diabetic medication adherence in recent years. This signifies broader awareness of non-adherence as an important issue in diabetes therapy, and an ongoing effort to find interventions that could improve adherence to anti-diabetic medications in patients with T2D.

Whilst most of the studies identified were conducted in the USA, there were a number of studies conducted in a broad range of countries. Diabetes is a global problem [2], with an increasing prevalence. The management of diabetes is influenced by social, cultural and environmental practices [77, 78]. It is therefore important that intervention studies are conducted in diverse populations globally to gain a better understanding of how to influence the range of factors that impact adherence to anti-diabetic therapy.

Characteristics of the subjects for enrolment into the study differed across the intervention studies. The reasons for apparent inconsistency in the subjects’ age groups is difficult to comprehend, especially as ‘age’ has been recognized, although inconsistently, as a factor that could affect adherence [7]. It is possible that the variations in age range, and the number of subjects recruited across different age brackets, have an impact on the effectiveness of the interventions in improving adherence to anti-diabetic medications, which may not have been detected or taken into account during the analyses.

In the same way, criteria set for HbA1c level varied across studies and the basis of demarcation was inconsistent and unclear. Ideally, the target HbA1c levels for most people with diabetes is recommended to be <7% to reduce the incidence of complications, particularly microvascular disease [4]. A better judgment of the impact of any intervention on glycaemic outcome is likely to be derived from patients who have high HbA1c levels, rather than ones who are already within the recommended range.

The duration since diagnosis and therefore the period of time that the patient has had diabetes, is likely to impact on patients’ self-management behaviours [79, 80], including medication adherence. For example, for all chronic diseases, it has been found that the first year after starting chronic medication is the period of highest risk for non-adherence [81]. While some studies have clearly defined the ‘duration of disease’, many have not. Therefore, consistency in sample selection, and inclusion of a clearly defined sample population with comparable traits could yield a better interpretation of the findings. Alternatively, a larger sample size can be recruited and the impact of characteristics such as duration of diagnosis, age and other variables as confounders can be determined.

Most of the studies in the review either used a control or comparison group or a pre/post research design. A randomized controlled trial is regarded as the most powerful research design [82], and most of the studies reviewed were randomized controlled trials. However, despite having the control group, some studies reported the impact of the intervention on the study group using pre and post-intervention data rather than comparing with the data from the control group, or they compared the effect size between the study and control groups without conducting appropriate statistical analyses to consider the change in outcome measures in the control groups. Therefore, it is not possible to determine the true impact of the intervention on adherence to anti-diabetic medication in these studies.

The setting in which the intervention is delivered is likely to have an influence on the delivery and impact of the intervention. For example, a noisy, crowded place may be considered unsuitable for effective delivery of educational sessions or counselling a patient. Most interventions in the review were conducted in community settings, and information about the settings and the environment of intervention delivery was not provided in any of the studies. It is therefore not clear how the setting and environment impacted intervention delivery.

### Medication adherence

Adherence to anti-diabetic medications was assessed as a primary outcome in most studies, using mainly self-report as the method of assessment. Although there was consistency in the use of the term adherence or compliance, the definition and interpretation of medication adherence was variable. For most studies, ‘taking or not taking the medication’ was the basis of the adherence measure, while others used number of patients reporting/not reporting a missed dose. To add complexity to the adherence definition, studies tended to use a cut-off point at which they considered patients as adherent or non-adherent, usually '≥80%', for example MPR ≥80%, and number of patients who were adherent to ≥80% of their OHA doses. In another study, where adherence percentage was tabulated, the range '0–25% adherent' was interpreted as non-adherent, while '50–100%' as adherent. In most of the studies the method/tool used for measuring medication adherence was the only clue to the definition/interpretation of adherence. For example, for those studies that used the SDSCA question, 'medication adherence' was defined as ‘the number of days in the past week the patient took their medication’. Therefore, with each different measurement approach used, the interpretation of 'medication adherence' varied. Thus, lack of a consistent approach in defining and interpreting medication adherence is apparent, preventing comparison between studies.

Furthermore, adherence to anti-diabetic medications was measured collectively, i.e. adherence to the whole therapeutic class, rather than to an individual anti-diabetic medication. Patients with T2D often take more than one medication for their diabetes, and whether or not adherence to each medication differs, needs to be assessed, particularly when comparing oral agents with injection therapy. Addressing medication adherence for each medication a patient is taking, could also be a way of determining patients’ perceptions of their individual medications and investigating whether the medication itself has an impact on adherence, and whether this impact varies depending on the total number of medications taken.

A range of tools are available to measure medication adherence, each having its own pros and cons [83]. The choice generally depends on the ease of use, validity and reliability [84]. Overall, self-report tools were the more common tools used in the studies reviewed, as seen in most adherence studies [85]. Self-report tools are popular because of their flexibility, ease of use, cost effectiveness and ability to gather social, situational and behavioural data [85]. SDSCA was the most commonly used self-report tool to estimate adherence in T2D patients. In addition to the inherent advantages that SDSCA has as a self-report tool, including being brief, reliable and valid for use in both research and clinical practice [76], its popularity can be explained by the fact that medication adherence in patients with T2D can be assessed as an outcome together with other important outcomes, for example adherence to diet, medication, blood glucose testing, lifestyle, foot care, and attending clinics. SDSCA allows assessment of the overall treatment adherence behaviours in patients with T2D, while also making it possible to assess adherence to a single component, using a single tool. Despite SDSCA remaining a popular method for measuring medication adherence, other self-report tools were also used to assess medication adherence, particularly after 2008, either alone, or in combination with another self-report tool or a different method. Specifically, MARS, MMAS, BMQ, and Hill Bone compliance scale, which is a scale generally used to measure adherence in hypertensive patients, were used, signifying the availability of a range of adherence measures and the lack of a universal measure.

Studies that used more than one method to assess medication adherence, either employed a second self-report tool, or a different method, for example electronic measure. Use of an additional method of measuring adherence was more common in studies published after 2008. Using two or more different methods could be an important means of validating, and/or triangulating the data, leading to improvements in medication adherence data collection. Nonetheless, a careful evaluation of the results obtained from both measures is required. For example, in two studies, where MEMS and self-report were used to assess medication adherence, although the trend appeared consistent for the intervention groups (from both measures), results for the control groups varied. The control groups, in each case, reported a better medication adherence based on the data from the self-report tool. Therefore, whilst it’s important to use more than one adherence measure to triangulate the data collected, these findings highlight the need to determine the aspect of adherence (initiation, compliance, persistence) being measured by each tool, as well as the tools’ characteristics, when choosing the tool and interpreting the data.

Diversity in the interpretation of the term 'medication adherence', methods of assessment, and reporting of the assessment results, thus, makes it very difficult to bring together the findings of all of the retrieved studies to make an effective comparison and conclude on the impact of interventions to promote adherence to anti-diabetic medication therapy. Moreover, a key aspect of intervention studies aimed at improving adherence is the appropriateness and validity of the methods and tools employed to determine the impact of the interventions.

### Intervention

A large number of interventions identified in the review failed to show a positive impact on medication adherence. Of the small number of ‘successful’ interventions, a few employed similar strategies that were also reported to be unsuccessful in improving medication adherence in other studies. It is therefore extremely difficult to conclude and predict what type of interventions will be the most effective in promoting adherence to anti-diabetic medications. While 65.4% of the total studies assessed the impact of their intervention on HbA1c, about half of these reported improvements in HbA1c, and only about a quarter reported improvements in both HbA1c and medication adherence. Improving HbA1c is the key in diabetes control, and all management efforts are aimed at bringing HbA1c levels to an acceptable range. Studies have indicated that adherence to therapy results in better controlled HbA1c [9, 10, 86]. However, this could not be confirmed from the studies included in the review, as the results obtained from studies which assessed an impact on both medication adherence and HbA1c, were inconsistent. Some improved medication adherence only, some HbA1c only and some improved both. Such inconsistencies lead us to question the level of adherence to anti-diabetic medications required in order to improve HbA1c levels; it also necessitates a clearer understanding of the role of other self-care measures (e.g. adherence to diet and exercise) in impacting on the HbA1c value, independently of, and together with, medication adherence. Anti-diabetic medication adherence could therefore be only one factor amongst a range of factors that contributes to the glycaemic control in T2D patients. A clearer understanding of how much being adherent to medication counts in terms of effective glycaemic control will help to guide effective patient education.

To understand the relative “weightings” of adherence to medications and self-care behaviours in impacting and improving HbA1c levels, it is important to have a better understanding of how each intervention (and its components) influences medication adherence and self-care behaviours. Most interventions in the studies have been designed with an intention to influence patients’ adherence to more than one self-care behaviour rather than just ‘medication adherence’. Some studies were able to impact one or more self-care behaviours, even if they did not have a positive impact on anti-diabetic medication adherence. A specific behaviour might be particularly susceptible to certain influences, for example, dietary behaviour is particularly susceptible to social influences [87], and might require a different approach to create a change. Quantifying the level of impact of an intervention on adherence to medication and other self-care behaviours will provide valuable information on the type of intervention that would be beneficial in not only improving medication adherence and adherence to a range of self-care behaviours, but also in improving and reaching HbA1c goal levels.

The majority of interventions reported an impact on one or more outcomes assessed, either clinical or non-clinical for example, adherence to foot care, adherence to blood glucose testing, patient knowledge, quality of life, blood glucose levels and lipid profiles. Thus, although most of the interventions did not improve anti-diabetic medication adherence or HbA1c, the intervention(s) improved other important patient-related outcomes, highlighting that each of the interventions could have one or more characteristics crucial to improving outcomes important in overall diabetes care.

The evidence presented in this review, therefore, suggests that a single strategy may not be suitable to effectively address all self-care behaviours and HbA1c levels in patients with diabetes. However, it may also not be practical to have several interventions each aimed at influencing different patient-related outcomes, including medication adherence. Additionally, it is equally important to be able to quantify the relative importance and weighting of the different self-care measures in impacting HbA1c in diabetics. This could help both the carers and the patients to better understand the role of medication related and non-medication related self-management strategies in patients with T2D. Thus, an ideal intervention would be one that has specific and targeted strategies addressing all key factors and which can be tailored to each patient, to specifically address the individual issues that impact medication adherence and glycaemic control.

### Conclusion

Most of the interventions identified in the reviewed studies aimed to influence a range of self–care behaviours, including medication adherence as well as clinical/non-clinical outcome(s). Interestingly, almost all interventions were successful in impacting one or more of the outcomes analysed, thus signifying that while the search for an effective strategy to influence adherence to anti-diabetic medication is yet not over, the interventions, and/or their components could be utilized singly or in combination to impact different clinical or non-clinical patient outcomes. While an ideal perspective would demand a single strategy, there is no evidence to support an effective single intervention to promote adherence to anti-diabetic medication(s) for all patients.

The intervention studies have used different methods to assess anti-diabetic medication adherence, with self-report tools being the most common. The tools have evolved over the years, and although SDSCA remained the most commonly used self-report tool, more recent studies have employed other novel approaches. Accurate assessment of medication adherence is critical for the correct interpretation of the effect of interventions on medication adherence. Clarity in the definition of medication adherence, and how it is measured are the issues that researchers have to consider when designing and implementing interventions.

It is evident from the review that although numerous strategies have been utilised to improve anti-diabetic medication adherence in patients with T2D, only few have succeeded, and even fewer have had an impact on HbA1c, the major indicator of glycaemic control. The successful interventions, akin to the overall studies in the review, employed variable strategies and methods of assessing medication adherence; it is therefore not possible to deduce the best possible strategy to address anti-diabetic medication adherence in patients with T2D.

## Limitations of the review

One of the limitations of this review was the heterogeneous nature of the studies included, which is inherent in adherence intervention studies and a fundamental issue that needs to be addressed in this area. The diversity in methods and measures used prevented a meta-analysis from being conducted. A further limitation is that as part of the review we did not consider the quality of the research design as an inclusion/exclusion criterion as we were interested in including all studies which had implemented and evaluated interventions to promote adherence to anti-diabetic therapy in T2D.

## Supporting Information

S1 PRISMA Checklist PRISMA Checklist. S1_Checklist.doc
(DOC)

S1 Table Review inclusion and exclusion criteria. S1_Table.docx
(DOCX)

S2 Table Operational definitions. S2_Table.docx
(DOCX)

S3 Table Study Characteristics. S3_Table.docx
(DOCX)

S4 Table Medication Adherence Assessment. S4_Table.docx
(DOCX)

S1 Fig Literature review search strategy. S1_Figure.docx
(DOCX)

S2 Fig Citation selection flowchart for the review. S2_Figure.docx
(DOCX)

S3 Fig PRISMA 2009 Flow diagram. S3_Figure.doc
(DOC)

S1 Appendix Full electronic search strategy for Medline database. S1_Appendix.docx
(DOCX)

S2 Appendix Intervention Summary and Impact. S2_Appendix.docx
(DOCX)
