# Supplementary material for: A Systematic Review of Interventions Addressing Adherence to Anti-Diabetic Medications in Patients with Type 2 Diabetes—Impact on Adherence
Source: PLoS One. 2015 Feb 24;10(2):e0118296. doi: 10.1371/journal.pone.0118296 (PMC4339210; doi:10.1371/journal.pone.0118296)
Supplement: S1 Table — S1_Table.docx (DOCX) [file pone.0118296.s002.docx]

**Table S1: Review inclusion and exclusion criteria**

|  | **Inclusion criteria** | **Exclusion criteria** |
| --- | --- | --- |
| Study Objectives | Program/ trials or intervention studies in patients with T2D that have evaluated the impact of the intervention on adherence to anti-diabetic medications, as primary or secondary outcome measure | Program/ trials or intervention studies in patients with T2D, that have evaluated the impact of the intervention on self- care measures or other diabetic outcomes without assessing the impact on adherence to anti-diabetic medications |
| Medications included | Studies including co-morbid condition(s) and other medications in patients with T2D, if:   - impact of intervention on adherence to anti-diabetic medications is explicitly presented and discussed | Studies including co-morbid condition(s) and other medications in patients with T2D, if:   - impact of intervention on adherence to anti-diabetic medications is not presented separately; or - adherence to medication is presented without specifying the type/ group of medication |
| Type of diabetes | Studies including adult patients with Type 1 and Type 2 diabetes or addressing ‘diabetes’ in general, if:   - effect of intervention on adherence to anti-diabetic medication is separately analysed for the two groups - adherence is measured and explicitly reported for oral hypoglycaemic agents | Studies including patients with Type 1 and Type 2 diabetes or addressing ‘diabetes’ in general, if:   - effect of intervention on adherence to anti-diabetic medicine is addressed for the diabetic patients as a whole (Type 1 plus Type 2)   Studies only with Type 1 patients, or with children and adolescents (Type 1 and 2) |
| Other | Studies that addressed medication adherence in patients with T2D but did not explicitly state that adherence to anti-diabetic medication were assessed. | Studies where:   - Full text could not be retrieved - Not available in English language - Published before January 2000 and after April 2013 |

T2D = Type 2 diabetes
