# Supplementary material for: A Systematic Review of Interventions Addressing Adherence to Anti-Diabetic Medications in Patients with Type 2 Diabetes—Impact on Adherence
Source: PLoS One. 2015 Feb 24;10(2):e0118296. doi: 10.1371/journal.pone.0118296 (PMC4339210; doi:10.1371/journal.pone.0118296)
Supplement: S2 Table — S2_Table.docx (DOCX) [file pone.0118296.s003.docx]

**Table S2: Operational definitions**

| **Variable** | **Definition** |
| --- | --- |
| Study duration | Timeframe starting from baseline data collection to time of final assessment, or ‘study duration’ as mentioned in the article. |
| Study setting | Place / setting where patients received the intervention. The study settings have been categorised as:   1. ‘Community’: the study setting was considered a ‘community’ setting when: 2. the word ‘community’ was used to describe the setting for example: community health care centre, community centre, community pharmacy (excluding community hospital) 3. the setting was described as: church based, pharmacy, primary care centres/ practices, primary health care, primary 4. when the intervention involved a telephone call or text messages (SMS) delivered to patients and no specific setting was stated. 5. ‘Hospital’: when the setting was described as a ‘hospital’ including a ‘community hospital’ 6. ‘Clinic’: when the setting was described as a ‘clinic’ or ‘medical centre’ |
| Primary/ Secondary outcomes | 1. Assessment of medication adherence was categorised as a 'primary outcome', when: 2. the study clearly specified it as a 'primary outcome' 3. measuring adherence was the 'primary aim' of the study 4. medication adherence was assessed as part of self-care or self-management or behavioural outcomes, which the intervention intended to impact as its primary aim 5. medication adherence was not reported as a ‘primary’ or ’secondary’ aim but was clearly an outcome measure of the impact of intervention evaluated 6. Assessment of medication adherence was considered as a 'secondary outcome', when: 7. the study specified it as a 'secondary outcome' 8. when 'primary outcomes' were defined but did not include 'adherence' 9. when measuring adherence was not the main focus of the study |
