# Supplementary material for: A Systematic Review of Interventions Addressing Adherence to Anti-Diabetic Medications in Patients with Type 2 Diabetes—Impact on Adherence
Source: PLoS One. 2015 Feb 24;10(2):e0118296. doi: 10.1371/journal.pone.0118296 (PMC4339210; doi:10.1371/journal.pone.0118296)
Supplement: S3 Table — S3_Table.docx (DOCX) [file pone.0118296.s004.docx]

**Table S3: Study Characteristics**

| **Author(s)/**  **Year/**  **Country** | **Study design*** | **Study subjects** | **Study setting** | **Sample size**  Control (***C***); Intervention (***I***); Comparison group ***(CC)*** | **Study duration** | **Outcomes analysed*** |
| --- | --- | --- | --- | --- | --- | --- |
| Hendricks LE, Hendricks RT/  2000/  USA [24] | Cross sectional; pilot study | African American men with T2D | Community | Allocated:  ***CC1*:**  15; ***CC2***: 15  and,  Analysed:  ***CC1***: 15; ***CC2:*** 15 | 6 months | HbA1c; diabetes knowledge; perception of general health; low blood sugar reaction; compliance to medication, diet, exercise regimen and daily foot care |
| Grant RW et al/  2003/  USA [25] | Prospective Randomized Controlled Trial | T2D patients | Community | Allocated: ***C*:** 114;***I*:** 118andAnalysed: ***C:*** *58;* ***I:*** *62* | 3 months | Adherence to medication, diet, exercise; HbA1c; cholesterol; medication discrepancies |
| Kim HS, Oh JA/  2003/  South Korea [26] | Randomized design with control and experimental groups being assessed pre- and post-intervention | T2D patients with HbA1c levels >7%, | Community | Allocated :  ***C:***: 25; ***I:***.: 25  and,  Analysed:  ***C:***: 16; ***I:***.: 20 | 12 weeks | HbA1c;Adherence to diet, exercise, blood glucose testing, medication taking, hypoglycaemic management & foot care |
| Maddigan SL et al / 2004 /  Canada [27] | Controlled prospective pre and post- trial | Rural T2D patients with sufficient English literacy | Community | Allocated:  ***C:*** 183; ***I:*** 210  and,  Analysed  @ baseline:  ***C***: 172; ***I***: 200  @ 6- months:  ***C***: 144; ***I***: 178 | 6 months | Health related quality of life; self-efficacy, beliefs and attitude towards diabetes; satisfaction with care; adherence to diet, exercise, BG testing and diabetes medication |
| Rosen MI et al/  2004/  USA [28] | Randomized controlled trial | Patients prescribed metformin | Clinic | ***C***: 17 ; ***I***.: 16 | 28 weeks | Mean adherence to metformin; mean adherence to other anti- hyperglycaemic; HbA1c |
| Schectman JM et al/  2004/  USA [29] | Not defined | Diabetic patients assigned to the panel of study physicians, and having 2 or more refills of oral diabetic agents from the practice pharmacy | Hospital | 83 physicians were provided with the feedback;  44 attended at least 1 of the 5 sessions  Total of 340 patients : mean of 4.1 patients per physician (range 1- 17) | 6- months | Change in medication refill adherence |
| Wermeille J et al/  2004/  UK [30] | Prospective pre-test- post-test single group design | T2D patients aged >40 years and on oral hypoglycaemic therapy | Community | Allocated:62  and,  Analysed: 59 | 28 weeks | HbA1c, BP, Lipid profile; medication compliance; patient knowledge; addition or increase in dose of oral hypoglycaemic, anti- hypertensive, lipid lowering and aspirin medicines |
| Odegard PS et al/  2005/  USA [31] | Randomized controlled multi-clinic trial | T2D patients age ≥18 years on at least one OHA, with an HbA1c ≥9% | Clinic | Allocated  ***C***: 34 ; ***I***: 43  and,  Analysed:  @ 6 month:  ***C***: 30; ***I***: 39  @ 12 month:  ***C***: 27; **I**: 39 | 12 months | HbA1c; medication appropriateness; medication adherence |
| Keeratiyutawong P et al/  2006/  Thailand [32] | Randomized controlled trial | T2D (21- 60 years of age) for <10 years, on treatment only with oral medications, FBG >130 mg/dL at least 2 consecutive times before entering the trial | Hospital | Allocated:  ***C:*** 45; ***I***: 45  and,  Analysed:  ***C***: 41; ***I***: 40 | 6 months | Diabetes knowledge; Adherence to diabetes self- care activities (diet, exercise, self- monitoring, foot care and medication taking) ; Diabetes quality of life; HbA1c |
| Kim HS et al/  2006/  South Korea [33] | Quasi- experimental, 1 group, pre-test – post-test | T2D patients | Community | Allocated: 44  and,  Analysed: 33 | 12 weeks | HbA1c;  adherence to: diabetic diet, 30 minute physical exercise, diabetic medication taking and foot care |
| Vincent D et al/ 2007/  USA [34] | Two-group, prospective experimental design | Mexican American, aged between 18- 75 years of age with T2D | Clinic | Allocated: 20  and,  Analysed: 17  ***C***: 8; ***I***.:9 | 12 weeks | Diabetes knowledge; self- efficacy and self- management behaviours (diet, medication, exercise, blood glucose, foot care); and biological measures: blood glucose, HbA1c,  Weight and BMI |
| Faridi Z et al/  2008/  USA [35] | Pilot controlled trial | T2D patients > 18 years of age, diagnosed at least 1 year prior to study, controlled by diet or oral medications (not on insulin) for at least 3 months; BMI > 25; and HbA1c < 8% | Community | Allocated:  ***C***: 15; ***I***: 15  and,  Analysed  ***C:*** 15; ***I***: 15 | 3 months | Feasibility and utility in enhancing diabetes management of NICHE; BMI, BG, HbA1c;  Physical activity; Patient’s self- efficacy; and diabetes self- care activities (diet, exercise, BG testing, foot care, medications, smoking status) |
| Quinn CC et al/  2008/  USA [36] | Randomized controlled trial | T2D patients 18- 70 years of age with A1C ≥ 7.5% and on stable diabetes therapeutic regimen for 3 months | Community | Allocated: 30  ***C***: 15; ***I***. : 15  and,  Analysed: 26  ***C***: 13; ***I:*** 13 | 3 months | HbA1c; changes in medication, adherence to self- care measures: diet, medications, exercise; self –reported control issues |
| Utz SW et al/  2008/  USA [37] | Quasi- experimental design  (Pilot study) | African American patients resident of the rural county | Community | Allocated: 22  ***CC1***: 14  ***CC2***: 8  and,  Analysed: 21  ***CC1***: 13  ***CC2***: 8 | 6 month | Goal achievement; Daily actions for diabetes self -care (general diet, specific diet, exercise, blood glucose testing, foot care, medication administration, smoking); Self efficacy; HbA1c and Satisfaction with diabetes education |
| Babamoto KS et al/ 2009/  USA [38] | Prospective randomized pre- post measurement | Hispanic/ Latino diagnosed with T2D at least 6 months prior to the study enrolment | Multiple setting: community/ clinic / home as appropriate | Allocated:318 (total),  and,  Analysed:189 (total)  ***CC1***: 75; ***CC2***: 60; ***CC3***: 54 | 6 months | Behavioural characters viz. dietary habits, exercise, medication use, admission to emergency department, mean diabetes knowledge, good health etc.  Clinical measures: HbA1c, BMI |
| Clarke A/  2009 /  Ireland [39] | Correlational, longitudinal study | Newly diagnosed T2D patients | Clinic | Allocated: 168  and,  Analysed: 168 | 6 months | Diabetes attitude; nutrition self- efficacy, exercise self- efficacy, perceived social support, emotional/ informational support, tangible support, affection support, positive interaction support; self- management: dietary, physical exercise, medication adherence behaviour |
| Glasgow RE et al/  2009/  USA [40] | Hybrid preference/ randomized controlled design | T2D patients aged 18 or older (members of the Kaiser Permanente Colorado Health Plan) | Community | Allocated: 198  and,  Analysed: 155 | 6 months | Healthy eating days, physical activity days, medication adherence, blood glucose testing, diabetes distress, patient activation, self- efficacy, problem solving, HbA1c, LDL cholesterol, Systolic BP |
| Kolawole B et al/  2009/  Nigeria [41] | Cross- sectional comparative study | T2D of Obafemi Awolowo University Teaching Hospital, Ile- Ife, Nigera, previously registered as members of the Diabetes Association of Nigeria (DAN) | “Undefined”  Reports that program is organized as part of statutory activities without specifically mentioning where it is held | Allocated:  ***CC:*** 75; ***I:*** 75  and,  Analysed:  ***CC***: 75; ***I***: 75 | Not specified | Knowledge of diabetes; Compliance; Monitoring devices; Hypoglycaemia; Treatment Satisfaction |
| Mullan RJ et al/ 2009/  USA [42] | Pilot, cluster randomized trial | T2D for at least 1 year, HbA1c between 7% – 9.5% taking 3 or fewer OHA and not using insulin | Community | Allocated:  ***C:*** 37; ***I:*** 48  and,  Analysed:  ***C***: 31; ***I***: 41 | 6 months | Acceptability of the decision aid; knowledge, decisional conflict and trust; patient involvement in decision making; medication decision; medication adherence |
| Rodin HA et al/  2009/  USA [43] | Quasi- experimental pre- post design | Diabetes subpopulation (members of diabetes related treatment and pharmacy claims: a prescription was filled for sulfonylurea, metformin, thiazolidinedione or insulin) | Community | Medical and pharmacy claims were analysed in:  ***C:*** 4089; ***I:*** 1846 | 4 years  Pre-and post-period, and consisted of 2 years each | Effect on Adherence and medical care expenditures |
| Sacco WP et al/ 2009/  USA [44] | Randomized pre- post study | Age: 18- 65 years; HbA1c greater than 6.5% | Community | Allocated:  ***C***: 31; ***I***: 31  and,  Analysed:  ***C***: 27; ***I***: 21 | 6 months | Adherence to self- care activities (diet, exercise, glucose testing, medication and frequency of feet inspection) ; BMI; HbA1c; Diabetes related medical symptoms; Depression Symptoms; Knowledge and understanding; Self efficacy; social support from the health care team; reinforcement for self- care activities; awareness of self- care goals |
| Thoolen BJ et al/  2009/  the Netherlands [45] | Prospective randomized controlled trial | T2D patients, newly diagnosed (3- 33 months of diagnosis) | Community | Allocated:  ***I:***108; ***C:*** 89  and,  Analysed:  ***I:***102; ***C:*** 78 | 12 months | Proximal outcomes like intentions, self- efficacy &proactive coping; and self- care behaviours viz. diet, exercise &medication adherence |
| Adepu R, Ari SM / 2010 /  India [46] | Prospective, randomized, interventional study | Patients with T2D, or HTN or both | Hospital | Allocated (total sample): 240  and,  Analysed (total sample): 227  (Note: Sample size not specified for Diabetes groups in particular) | 3 months | Medication adherence; knowledge, attitude &practice behaviour; blood pressure (in patient groups with HTN & HTN+DM and capillary blood glucose |
| Bogner HR, de Vries HF /  2010 /  USA [47] | Pilot randomized controlled trial | African American T2D patients ≥50 years; HbA1c >7% or a prescription of OHA within the year before the study and a diagnosis of depression or a prescription for an antidepressant within a year before the study | Community | Allocated:  ***I***: 29; ***C:*** 29  and,  Analysed:  ***I:*** 29; ***C:*** 29 | 12 weeks | HbA1c ; Depression scores; adherence to OHA & antidepressants |
| Borges APDS et al/  2010/  Brazil [48] | Prospective, experimental, controlled study | T2D patients ≥ 18 years of age | Community | Allocated:  **C:**31; ***I:*** 40;  and,  Analysed:  ***C:*** 31; ***I:*** 33 | 12 months | Clinical parameters:  Fasting glycaemia, HbA1c, Framingham Cardiac Risk Scores; adherence to therapy; number of drug therapy problems identified and solved |
| Castillo A et al/  2010/  USA [49] | Non experimental pre-test post- test single group (pilot) study | Self- reported T2D Hispanics/ Latino patients, age 18 years and older | Community | Allocated: 70  and,  Analysed: 70 | 10 weeks | Diabetes knowledge, self- care behaviours, self- efficacy, depression, medication taking behaviour, HbA1c, weight, blood pressure |
| Cinar FI/  2010/  Turkey [50] | Single centre, prospective study | T2D patients with HbA1c ≥6.5% | Community | Allocated: 35  and,  Analysed: 35 | 3 months | Adherence to diet, exercise, drug; metabolic parameters: HbA1c, FPG, PPBG, TG, LDL, HDL, BMI,SBP and DBP |
| Gonzalez JS et al/  2010/  USA [51] | Case series analysis  (pre- post assessment) | “Diabetic (sub-optimally controlled) &depressed” from Massachusetts General Hospital Diabetes Clinic | Hospital | Allocated: 5  and,  Analysed: 5 | 4 months | Adherence to OHA, HbA1c, Glucose monitoring and other self -care activities (SDSCA)and depression measures |
| Tang TS et al/  2010/  USA [52] | Control- intervention time series design with subjects serving as their own controls | African- American with T2D living in the greater Ypsilanti, Michigan area | Community | Control and intervention are the same sample;  Allocated= 77  and,  Analysed: 77 | 12 months | HbA1c, lipids, blood pressure, weight & BMI, LDL & HDL  Diabetes specific QoL; Self- care behaviours (healthy diet, spacing carbohydrates, exercising, monitoring blood glucose, inspecting feet, taking medication, using insulin); and Empowerment |
| Wolever RQ et al /  2010/  USA [53] | Randomized controlled trial | T2D patients at least 18 years of age, diagnosed for at least 1 year, be taking oral diabetes medications for at least 1 year | Community | Allocated : 56  ***C***: 26; ***I***.: 30  and,  Analysed: 49  ***C***: 22; ***I***: 27 | 6 months | Change in scores of the following outcome measures, for eg.: ASK- 20, Morisky scale, Patient Activation Measure- 13 (PAM- 13), Appraisal of Diabetes Scale, Interpersonal Support Evaluation List (ISEL- 12), Perceived stress scale (PSS-4), Short- Form Health Survey (SF- 12), Benefit finding scale (BFS); Exercise, HbA1c |
| Zhang Y et al/  2010/  USA [54] | Quasi- experimental pre- post design, with 3 treatment groups and a comparison group | Older adults, 65 yrs or older with diabetes* | Community | ***I:***  No coverage group: 247  $150- cap group: 304  $350- cap group:2214  and,  **CC:**  No cap group: 1253 | 3 years  (study period through 2004 to 2007) | Change in Medication Possession Ratio (MPR), Likelihood of good adherence and treatment intensity |
| Gracia- Huidobro D et al/  2011/  Chile [55] | Controlled clinical trial | T2D patients between 18- 70 years of age, with an HbA1c≥7% in previous 3 months  and living in the same household with a significant family member >15 years of age | Clinic | Allocated:  ***C1***: 76; ***C2***: 84;  ***I***: 83;  and,  Analysed:  @6 months  ***C1***: 76; ***C2***: 84;  ***I***: 83  @ 12 months:  ***C1***: 76; ***C2***: 84;  ***I***: 83 | 12 months | HbA1c ;  Health behaviours: Breads per day , Use of sugar , Fruits or vegetables per day,  Physical activity (number of days per week), Medication adherence, Family functioning style, Knowledge of diabetes  Presence of depressive symptoms |
| Khan MA et al/  2011/  USA [56] | Randomized, controlled trial | T2D patients of ≥18 years of age, responsible for their own diabetes self- management uninsured, primarily ethnic minority adults | Clinic | Allocated:  ***C***: 62; ***I***.: 67  and,  Analysed:  ***C***: 47; ***I***.: 53 | 3 months | Diabetes knowledge, diabetes self -efficacy, Medication adherence, SDSCA measures; HbA1c, BP, Number of BP medications, no. of oral diabetes medications, Insulin use |
| Mehuys E et al/  2011 /  Belgium [57] | Randomized, controlled, parallel group trial | T2D patients age 45- 75 years, BMI ≥ 25kg/m^2^, on OHA for at least 12 month | Community | Allocated:  ***C***: 135; ***I***.: 153  and,  Analysed:  ***C***: 132; I.: 148 | 6 months | Fasting plasma glucose; HbA1c; Adherence to OHAs; knowledge about diabetes; self- management and sustainability of study results |
| Mitchell B/  2011/  Australia [58] | Non- randomized, multi-centre, pre- post study | T2DM patients who had an HbA1c > 7.0% confirmed by their GP | Community | Allocated: 524  and,  Analysed: 323  (Note: No. of patients in whom medication adherence has been analysed is different, i.e. 345 to 347) | 6 months | Process measures:  Number of : self- management support interventions (SMSIs), goals set by the patients, of goals attained by the patient  Outcome measures:  Mean blood glucose level at each visits, Medication adherence, BMI, Physical activity, Smoking status Patient satisfaction |
| Piette JD et al/  2011 /  USA [59] | Randomized controlled trial | At least 21 years old T2D patient with significant depressive symptom | Community | Allocated :  ***C***: 167; ***I***: 172  and,  Analysed:  ***C***: 146; ***I***: 145 | 1 year | HbA1c, Blood pressure, Health Related QoL, step counts, Beck depression inventory, coping orientation, perceived competence, medication adherence, beliefs about medication |
| Ramanath KV, Santhosh YL /  2011/  India [60] | Prospective randomized controlled study | T2D patients | Hospital | Allocated:  ***C***: 56; ***I:*** 57  and,  Analysed:  ***C:*** 48; ***I***: 52 | 3 months | Medication adherence, blood glucose levels, Knowledge, Attitude and Practice (KAP), Quality of life |
| Shetty AS et al/  2011/  India [61] | Randomized  pilot study | T2D; age group 30- 65 years and with a minimum 5 years of diagnosis; HbA1c between 7% - 10% | Community | Allocated:  ***C***: 105; ***I***: 110  and,  Analysed:  ***C***: 66; ***I***: 78 | 1 year | Adherence to Physical activity, dietary and medicine;  BMI, Blood glucose level, HbA1c, TC, TG, HDL-C & LDL-C;  Acceptability of SMS |
| Smith SM et al / 2011/  Ireland [62] | Pragmatic cluster randomized controlled trial | Type 2 diabetes above 18 years of age | Clinic | Allocated:  ***C:***203; ***I:*** 192  and,  Analysed:  ***C***: 171; ***I***: 166 | 2 year | HbA1c, BP, cholesterol, wellbeing; BMI, self-care activities; self- efficacy; adherence to medications; family & friends subscale of chronic illness survey; smoking; measures of process of care |
| Wakefield BJ et al / 2011/  USA [63] | Single- centre, randomized, controlled clinical trial | T2D with HTN being treated by a VA primary care provider | Clinic | Allocated:  ***C***: 107; ***I1***: 93; ***I2***: 102  and,  Analysed:  @6 month:  ***C***: 97; ***I1***: 77; ***I2***: 83  @12 month:  ***C***: 94; ***I1***: 73; ***I2***: 79 | 12- month | HbA1c, Systolic BP  Adherence to anti-diabetic medicines and to anti- hypertensive medications |
| Walker EA et al /  2011/  USA [64] | Randomized controlled intervention | Adults (≥30 years of age) members of the health care worker union Fund based in New York City with prescription of at least one oral glucose lowering agent in the year prior to enrolment and A1c ≥ 7.5% | Community | Allocated: 527  and,  Analysed: 526 | 1 year | Change in HbA1c , pharmacy claims data; self-reported medication adherence measures and other self- care behaviours |
| Barron JJ et al/  2012/  USA [65] | Retrospective, observational cohort studies [discusses 2 studies] | Patients with at least 1 medical claim for diabetes or at least 1 pharmacy claim for an anti- diabetes agent | Community | Study 1 (PA):  I: 715  C: 497  Study 2(TDES):  I: 237  C: 237 | Study 1:  18 months  Study 2:  3 years (data evaluated) | Medication adherence; Diabetes related economic outcomes, such as Diabetes resource utilization and costs, Use of services for comprehensive diabetes care, and Utilization and costs of all health care services, regardless of diagnosis |
| Bogner HR et al/ 2012 /  USA [66] | Randomized controlled trial [a 2 week run in phase initially to collect pre-intervention adherence rates and other baseline data] | T2D patients aged ≥ 30 years with a current prescription of an OHA and a current prescription for an antidepressant | Community | Allocated:  ***C***:: 88; ***I***.: 94  and,  Analysed:  ***C***: 88; ***I***.: 92 | 12 weeks | Adherence to OHA and to anti-depressants;  HbA1c (change from baseline and achieved HbA1c <7%)  Remission of Depression |
| Brennan TA et al/ 2012/  USA [67] | Prospective cohort pre- post analysis | Patients with diabetes defined as people at least age 40 who were continuously eligible for pharmacy benefit management and who had filled a prescription for a medication to treat diabetes within 6 months of the program’s start | Community | ***I:*** 5123 patients- employees and covered dependents with diabetes  ***C***: 24124 people with diabetes, based on a roughly matched criteria | 6 months | Change in adherence to medications used to treat diabetes;  Initiation rate of concomitant therapies (statins and angiotensin receptor blocker) |
| Choi SE, Rush EB /  2012/  USA [68] | Single group pretest and post- test design (pilot study) | T2D patients 21- 80 years of age and have had diabetes for at least a year | Community | Allocated: 53  and,  Analysed: 41 | 3 months | Diabetes management: Diet, Exercise, Glucose checks, feet checks, Medication adherence  Health and well- being: Diabetes Knowledge, Diabetes self- efficacy, Mood, Health status  HbA1c, SBP, DBP, TC, LDL, HDL, TG, BMI, Waist circumference, Waist/ Hip ratio |
| Farmer A et al/  2012/  UK [69] | Parallel group randomized trial | T2D patients of at least 3 months duration, currently taking any OHA and with HbA1c ≥ 7.5% | Community | Allocated:  ***CC:*** 85; ***I***: 126  and,  Analysed:  ***CC:*** 81; ***I***: 114 | 20 week | The percentage of days over a 12- week period on which the correct number of doses of main glucose lowering medication was taken each day as prescribed;  Functional status, treatment satisfaction, satisfaction with the nurse delivering the intervention and the medication adherence |
| Kroese FM et al/  2012/  the Netherlands [70] | Not defined | T2D patients below the age of 75 years with sufficient conduct to Dutch language | Community | Initial phase: 129 patients  Dropout at:  Session 1: 1  Session 2: 9  Session 3: 17  Session 4: 56  So, total 56 patients dropped out | 6 months | Attendance and evaluation to the booster sessions  Effectiveness of the booster session assessed based on: diabetes self- care activities, Medication adherence, Lifestyle adherence, Physical activity, Dietary habits |
| Mellitus Janice C-M et al/  2012/  USA [71] | Pilot study , intervention study with both quantitative and qualitative measures | African American T2D patients in the southeastern United States | Community | Allocated: 12  and,  Analysed: 12 | 12 weeks | Self- care practice measures; perception of their current state of diabetes self- management; depressive symptoms; anxiety symptoms; general anxiety level; perceived stress; HbA1c, TC, LDL, HDL, BP, BMI |
| Odegard PS, Christensen DB/ 2012/  USA [72] | Randomized, multi-pharmacy, controlled trial | T2D adults (>18 years) receiving care at urban community pharmacies who are late receiving an oral diabetic prescription refill by 6 days or more | Community | Allocated:  ***C:*** 145; ***I:*** 120  and,  Analysed:  ***C:*** 145; ***I***. : 120 | 12 months | Changes in Medication Possession Ratio  Comparison of intervention effects on adherence for patients with low versus high MPR and comparison amongst Metformin and Sulfonylurea |
| Ramanath KV et al/ 2012/  India [73] | Prospective randomized study | T2D patients on medication for 6 months/ 18 years and above | Hospital | Allocated: 51  and,  Analysed:  ***C***: 24; ***I:*** 24 | 8 months | Blood sugar (fasting and post prandial); Medication adherence; Quality of Life; Patient satisfaction |
| Vervloet M et al/  2012/  the Netherlands [74] | Randomized controlled trial | T2D (18- 65 years) patients on anti-diabetic medication for at least 1 year with suboptimal levels of adherence (pharmacy refill rate of their OHA less than 80%) | Community | Allocated:  ***C:*** 208; ***I:*** 207  and,  Analysed:  ***C:*** 48; ***I:***56 | 6 months | Adherence to OHAs:  Number of days without dosing, Proportion of missed dose, Proportion of doses taken within agreed and predefined standardized time windows;  Patients response to SMS, reminders (impact of SMS) and patients experience with RTMM and with SMS reminders |
| Zolfaghari M et al/  2012/  Iran [75] | Quasi- experimental, two- group, pre and post- test; randomized | T2D patients on OHAs only recruited from Iranian Diabetes Association | Community | Allocated:  ***CC1***: 39; ***CC2***: 41  and,  Analysed:  ***CC1***: 38; ***CC2***: 39 | 6 months  (Note: only 3 months’ data has been presented) | HbA1c and adherence to diabetic diet, physical exercise and diabetic medication taking |

*terms used are as used in the (individual) study
