# Supplementary material for: A Systematic Review of Interventions Addressing Adherence to Anti-Diabetic Medications in Patients with Type 2 Diabetes—Impact on Adherence
Source: PLoS One. 2015 Feb 24;10(2):e0118296. doi: 10.1371/journal.pone.0118296 (PMC4339210; doi:10.1371/journal.pone.0118296)
Supplement: S4 Table — S4_Table.docx (DOCX) [file pone.0118296.s005.docx]

**Table S4: Medication Adherence Assessment**

| **Study** | **Medication Adherence as Primary or Secondary outcome?** | **Medication adherence measurement details**  **Method**  **Tool**  **Calculation and definition (where relevant)** | **Point of reference for assessing the adherence** | **Reported adherence result @ baseline** | **Reported adherence result post intervention** |
| --- | --- | --- | --- | --- | --- |
| Hendricks LE, Hendricks RT, 2000 [24] | Primary | Self- report  Tool: not specified  Tabulated as:  **Medication compliance** (Yes/ No), which is presented as n(%), where 'n' is defined as the ‘number of responses’ rather than the actual number of participants** | "Number of responses" | Not reported | Number of responses [n(%)] to medication compliance::  @ Monthly follow up:  Yes: 44 (94) / No: 3 (6)  @Follow up every 3 months:  Yes: 28 (93)/ No: 2 (7) |
| Grant RW et al, 2003 [25] | Primary | Self –report  Tool: (‘adapted from’) SDSCA  Measured as:   - the number of adherent days out of the past 7 days - ‘patients not missing any doses of any diabetes related medication on that day’ considered adherent for the day’ | No. of days | ***I.*** :6.7±0.9days ***C***: 6.9±0.4 days | Change from baseline @ 3 months i.e. post intervention:  ***I.:***0.1±1 days  ***C:***0.1±0.4 days |
| Kim HS, Oh JA, 2003 [26] | Primary | Self –report  Tool: 2- item questionnaire developed by the researcher as part of thesis (unpublished)  [*Cronbach’s alpha, value 0.82)]*  Score: 0- 100:  "0= never do at all’ and  "100 = always do as prescribed"  Data presented as:  'Average score of the two components, with higher score indicating better adherence | Adherence measure score | Scores (Mean ± SD):  ***I:*** 78.4±32.6  ***C:*** 92.0±22.6 | Scores (Mean ± SD):  ***I:***94.4±14.4  ***C:*** 98.8±3.8 |
| Maddigan SL et al, 2004 [27] | Primary | Self -report  Tool: Self Care Activities (SDSCA) questionnaire [2- item for medication] | Adherence measure score | Baseline (z- score):  ***I***. 0.020±1.01  ***C***: -0.098±0.70  [adherence was assessed for those who used oral medication or insulin to manage their diabetes] | Mean change scores (95% CI) from baseline to follow up:  Unadjusted:  ***I***: 0.02 (-0.09 to 0.14)  ***C***: -0.09 (-0.25 to 0.07)  Adjusted:  ***I***: 0.11 (-0.07 to 0.28)  ***C***: 0.17 (0.004 to 0.34) |
| Rosen MI et al, 2004 [28] | Primary | Electronic Measure & Self – report  Tools:  Electronic Measure: MEMS  Self- report: the response of the patient to what percentage [or number] of the time the medications were taken within 2 h of the agreed times.  Definition (for MEMS assessment):  Adherence was defined as 'the number of doses taken within 2 h of the agreed-upon time divided by the number of prescribed doses.'  [Duplicate doses taken were counted as one dose taken correctly] | Adherence reported/ recorded and calculated | MEMS :  the adherence of both the control and intervention groups was approximately 60%  Self- reported: adherence at baseline was 88% for the sample as a whole | MEMS (extracted from graph):  ***I***.: Adherence (to metformin, for which the counselling was focused) gradually increased to 80% by week 16 but decreased gradually after the intervention was discontinued;  Self -reported adherence also showed similar pattern in intervention group. In self- report, control group reported higher adherence than the intervention group, unlike the MEMS report |
| Schectman JM et al, 2004 [29] | Primary | Pharmacy refills  Calculated as:  Prescription Refill rates = 'the number of days of therapy dispensed on all but the last prescription in this interval divided by the total number of days in this interval'  Data presented in percentage (%) value, higher ‘%’ indicating higher adherence | Mean adherence change for each physician panel | Refill adherence (%) calculated for the patients in each panel group:  ***I***:83.9%  ***CC***: 86.5% | Refill adherence (%) calculated for the patients in each panel group@post intervention:  ***I***: 86%  ***CC***: 85%  Mean absolute increase in adherence for patients whose physician did not attend the educational session was 0.2% versus, a significant mean increase of 4.2%* for patients whose physician attended the educational program (results adjusted for baseline level of adherence)  Analysis in 106 patients with baseline level of adherence <80%, patients of physician attending the educational session showed greater improvement in adherence than patients of non-attendees(17% vs 10%, p= 0.09) |
| Wermeille J et al, 2004 [30] | Primary | Self- report & Pharmacy refill  Tools:  Self- report: Using a visual analogue scale (0–100%) to indicate how well patients felt they had taken their medicines during the previous month.  Pharmacy refill: Using the Patient Medication Record (PMR) system in community pharmacy: date of refill, dosage & no. tablets registered at the pharmacy in the 24 weeks preceding patient inclusion in addition to the full study period.  Calculation (PMR):  The percentage compliance was calculated by dividing the no. of tablets dispensed by the no. of tablets prescribed for the assessed period.  [For, baseline compliance assessment, the last day was the ‘day of the last prescription dispensed before the initial interview; the first day was the ‘date of first prescription dispensed at least 24 weeks before the last day’.  &  For final compliance assessment, the last day was the ‘day of the last prescription dispensed before patient final interview, the first day was the date of first prescription dispensed at least 24 weeks before the last day.]  If necessary, the final assessment period could include the inclusion date and the previous week. | Adherence measures  (% compliance) | 1. Patient self- report (n= 59)  Mean (SD) [%]:  97.5 (5.4)  2. Community pharmacy PMR system (n= 39)  Mean (SD):  94.9 (12.6) | 1. Patient self- report(n= 59)  Mean (SD) [%]::  98.3 (3.7)  2. Community pharmacy PMR system (n= 39)  Mean (SD):  99.7 (14.2) |
| Odegard PS et al, 2005 [31] | Secondary | Self- report  Tool: '2 question recall technique' based on the response to the following: “*Taking medications on a regular basis can be difficult. Do you ever find it difficult to remember to take (insert medication name)?” If yes, then, “How many times over the last 2 weeks have you missed a dose?*”  ‘No. of patients who reported difficulty in remembering to take their medication’ was used in analysis | Number of patients | % of patients who reported difficulty in remembering to take their medications:  ***I*** : 56%  ***C*** : 35%  (p= 0.07) | Data not presented  States that 'intervention had no effect on improving adherence' |
| Keeratiyutawong P et al, 2006 [32] | Primary | Self- report  Tool: SDSCA questionnaire (translated in Thai)  The questions asked *“the no. of days to perform diabetes self-care activities during the previous 7 days”[Scored: 0-14]* | 'Mean medication taking score' (only for baseline)  and, the no. of patient who increased 'medication taking' | Mean medication taking score:  ***I***: 12.45 (SD=3.01)  ***C***: 12.00 (SD=2.84) | Mean SDSCA medication adherence score is not presented post intervention.  Stated that:  *'After completing the program at 6 months, 17.5% of the subjects in the intervention group decreased medication intake while the control group decreased by 7.3%.*'  *'40% of subjects in the intervention group and 49% in control group increased medication intake*.' |
| Kim HS et al, 2006 [33] | Primary | Self- report  Tool: SDSCA questionnaire (1- item)   - *‘how many of the last 7 days, did you take your recommended diabetic medication?’[scored: 0- 7]* | Adherence measure score | Adherence, d/ wk  (mean ± SD):  4.8±2.6 | Adherence, d/ wk  (mean ± SD):  5.9±1.9  Difference (post-test- pre-test) : 1.1±1.9* (p= 0.032) |
| Vincent D et al, 2007 [34] | Primary | Self- report  Tool: SDSCA questionnaire (1- item)   - *"the no. of days in past 7 days the recommended medications were taken” [Scored: 0- 7]* | Adherence measure score | Mean (SD):  ***I***: 5.00 (3.0)  ***C***: 4.38 (3.6) | Mean (SD)::  @ Post- intervention   - ***I***.: 6.13 (2.5) - ***C***: 5.25 (3.2)   @ 4- weeks post intervention:   - ***I*** : 5.44 (3.1) - ***C***: 5.13 (3.2) |
| Faridi Z et al,  2008 [35] | Primary | Self- report  Tool: SDSCA questionnaire (1- item)   - "*the no. of days in past 7 days the recommended medication were taken*”   *[Scored 0- 7]* | Adherence measure score | Mean(SD):  ***I***. 5.0 (2.8)  ***C***: 5.4 (2.5) | Differences within each group post intervention:  Mean(SD):  ***I***.: 0.1(0.7)  ***C***: 0.7(1.2)* [p<0.05 in control] |
| Quinn CC et al, 2008 [36] | Not mentioned as an objective of the study or stated however tabulated as changes in behavioural outcomes (diabetes self- care) pre and post intervention] | Self- report  Tool: SDSCA (1- item)   - *"the no. of days the patient has taken their medication in last seven days is used to assess medication adherence"*   *[Scored 0- 7]* | Adherence measure score | Mean no. of days over 1- week period:  ***I***: 5.92  ***C***: 6.3 | Mean no. of days over 1- week period:  ***I***: 6.64  ***C***: 6.75 |
| Utz SW et al, 2008 [37] | Primary | Self- report  Tool: SDSCA questionnaire (2- item)   - *Scored: 0- 14* | Adherence measure score | Scores [Mean(SD)]:  ***CC1:***12.09(4.53)  ***CC2***:12.6 (3.13) | Scores [Mean(SD)]:  ***CC1***: 13.56(1.33)  ***CC2***:12.29 (4.54)  Post- baseline changes in scores [Mean (SD)]:  ***CC1***: -0 .44(1.33)  ***CC2***: 1.0(2.24)  *[*Reports *the data were skewed & were virtually unchanged post intervention]* |
| Babamoto KS et al, 2009 [38] | Secondary | Self- report  Tool: "Morisky Self- Reported Behavioural Scale" (stated in methods), [but analysis done on one single response: ‘never forgetting to take their medications’]  No. of patients "Never forgetting to take their diabetes medications" is used in analysis | Number of patient | % of patients reporting to have never forgotten to take their diabetes medication:  ***CC1***: 69%; ***CC2***: 77% and ***CC3***: 67% | % of patients reporting to have never forgotten to take their diabetes medication after intervention:  ***CC1***: 79%; ***CC2:***55% and ***CC3***:, 50% |
| Clarke A, 2009 [39] | Primary | Self –report  Tool: Medication Adherence Report Scale [MARS] [Scored 5- 52]  Operational definition for adherence:  'self- reported habitual adherence to taking of prescribed medications' | Adherence measure score | Mean(SD):  23.33(2.79) | Mean(SD):  @1 month:  23.31(2.71)  @ 6 months  23.30(2.26) |
| Glasgow RE et al, 2009 [40] | Primary | Self- report  Tool: Hill- Bone Compliance Scale (medication - taking items)   - *Assessed '‘how often respondents missed medication due to a variety of reasons’* | Adherence measure score | Baseline value:  DVD only: 1.2 (0.04)  Class only: 1.1 (0.03) | Adjusted 6 months changes*:  DVD only: 0.0(0.02)  Class: 0.2(0.04) |
| Kolawole B et al, 2009 [41] | Primary | Self- report  Tool: Self- and interviewer- administered structures questionnaires (previously validated and tested) | No. of patients | Not measured | Significant difference *  Study group:  No. of patients reporting:  Poor compliance: 12 (16%)  Good compliance: 63 (84%)  Comparison group:  No. of patients reporting:  Poor compliance: 29(38.7%)  Good compliance: 46 (61.3%) |
| Mullan RJ et al, 2009 [42] | Primary | Self- report & Pharmacy refills  Tool:  Self –report: ‘adherence’ measured as the number of times the patient reported having missed taking their pills in the last week  Pharmacy refills: Evaluation of the pharmacy records of diabetic medications 6 months after their clinical visit  Definition (Pharmacy records):  “**Adherence**”: defined by the proportion of days covered during the 180 days after the visit, crediting overlapping supply.  “**Persistence**” : defined as the no. of days from first prescription fill to the last fill in the 180 days after the visit, giving credit for overlapping supply and the number of days supplied at the last fill, truncating at 180 days after the visit | 1.‘No. of patient who did not miss the dose’ is the point of measure for self- reported adherence analysis  2. No. of days for persistence and adherence for pharmacy refill record | Not reported | Patients who did not miss the dose in last week n(%):  ***I***: 31 (76%)  ***C***: 25 (81%)  Persistence over 180 days,  No. of days (range):  ***I:***180 (0- 180)  ***C:*** 180 (180- 180)  Adherence days covered,  % (range):  ***I***: 97.5 (0.0 to 100)  ***C***: 100 (73.9 to 100)  (control group was better) |
| Rodin HA et al, 2009 [43] | Primary | Pharmacy refills and prescription claims  Tool: Medication possession ratio   - MPR calculated by dividing the no. of drugs supplied by the no. of days the patient had the health condition of interest - MPR of at least 80% as adherent and, less than 80% as non- adherent | No. of patients were analysed and then change in adherence patient (%) was calculated | Not reported | Change in adherence (%)from pre- period to post period*:  Metformin:  ***C***: 3.1; ***I***.: 5.4  Sulfonylurea:  ***C***: 4.4; ***I***.: 5.0 |
| Sacco WP et al, 2009 [44] | Primary | Self- report  Tool: SDSCA questionnaire(2- item) | Adherence measure score | Not reported | Not reported |
| Thoolen BJ et al, 2009 [45] | Primary | Self- report  Tools:  Medication Adherence Report Scale, MARS (5- item),   - *measured the degree to which the patients forgot their medication, stopped taking their medication or changed dosage [Scored: ‘1’: always true to ‘5’: never true]*   &  SDSCA questionnaire (1- item)   - *[Scored 0- 7]* | Adherence measure score | MARS [Mean (SD)]:  ***I***: 4.8 (0.5)  ***C***: 4.9 (0.2)  SDSCA [Mean(SD)]:  ***I***: 6.9 (0.7)  ***C***: 6.9 (0.7) | MARS [Mean (SD)]:  @3 months:  ***I***: 4.9 (0.2)  ***C***: 5.0 (0.2)  @12 months:  ***I***: 4.9 (0.2)  ***C***: 5.0 (0.2)  SDSCA [Mean(SD)]:  @ 3 months:  ***I***: 6.9 (0.4)  ***C***: 6.9 (0.4)  @12 months:  ***I***: 7.0 (0.4)  ***C***: 6.9 (0.3) |
| Adepu R, Ari SM, 2010 [46] | Primary | Self- report  Tool: Brief Medication Questionnaire (BMQ)   - Consisting of **Regimen screen,** Belief screen , Recall screen, Access screen - Subtotal of each screen is **Adherence Risk Scale (ARS)** scored 0- 4 [0 = reports non adherence & 4= adherence] - Regimen screen accesses the medication adherence behaviour | Adherence measure scores | Control at baseline:  ARS: 1.11  Regimen screen: 1.04  DM test group:  ARS: 0.73  Regimen screen: 0.88 | Control at final:  ARS: 0.67  Regimen screen: 0.83  DM test group*:  ARS: 0.88  Regimen screen: 0.87  (the improvement was significant in all screen scores from baseline to follow up in the intervention group, but comparison against control has not been presented) |
| Bogner HR, de Vries HF, 2010 [47] | Primary | Electronic measures  Tools: MEMS   - Patient population who are ≥80% adherent to OHA is used in the analysis | No. of patients | Patient population who are ≥80% adherent to OHA:  ***C***: 6 (20.7%)  ***I***.: 10 (34.5%) | Patient population who are ≥80% adherent to OHA*:  ***C***: 7 (24.7%)  ***I***.: 18 (62.1%) [p=0.004] |
| Borges APDS et al, 2010 [48] | Secondary | Self- report  Tool: Morisky- Green test[4- questions]   - Scored: 0 to 4   Patients were considered adherent when they got a maximum score of four points, and non- adherent if they got 3 points or less | Adherence measure score | Average score at the start of the study for the intervention group at baseline is2.8  Data for several visits given for intervention group however no data presented for the control group | At the end of the study at 12 months, the mean score in intervention group was 3.9*  (the change at 12 month is significant compared to the baseline)  No data for the control group |
| Castillo A et al, 2010 [49] | Secondary | Self- report  Tool: SDSCA   - *the number of days in the week the patients reported taking their medication* | Adherence measure score | Mean (SD):  5.5(2.5) | Mean (SD)*:  6.6 (1.3) |
| Cinar FI et al, 2010 [50] | Primary | Self- report based on telephone interview?  In the intervention section says that: "*Medication compliance was assessed through the telephone interview, the patients were asked whether they used the drugs according to the prescribed form or not. Patient who did not know the name and the usage of any drug were considered inadaptable"* [which drug in particular was discussed was also not mentioned] | No. of patients | Patients reporting medication use adherence, n(%):  18 (51.4)  It is further reported that 'drug therapy non- compliance was found in 16 (45.7%) patients at the beginning ' | Patients reporting medication use adherence, n (%):  @Week 12*:30 (85.7) |
| Gonzalez JS et al, 2010 [51] | Primary | Electronic measure & Self report  Tool: Electronic measure: MEMS Self- report:   - the number of missed doses since the last study visit (used to supplement the MEMS), analysed as “% of the number of doses taken” (objective measurement by MEMS) - SDSCA questionnaire (1- item)   analysed as " “*the mean number of days per week the patient was adherent to their medication [Scored: 0- 7]* | Adherence measure score | MEMS report:93%, 100%, 57%, 93% & 100% for patient 1, 2, 3, 4 & 5 respectively  SDSCA scores: 6.7, 6.7, 4, 6 & 6 respectively for patient 1, 2, 3, 4 and 5. | MEMS report:  100%, 100%, 71%, 100% and 86% for patient 1, 2, 3, 4 and 5 respectively.  SDSCA scores: 6.7, 7, 5,7 & 6.3 for patient 1, 2, 3, 4 and 5 respectively |
| Tang TS et al, 2010 [52] | Primary | Self- report  Tool: SDSCA questionnaire (1-item)   - *'No. of days the patient took his medications in last 7 days' [Scored: 0- 7]* | Adherence measure score | Control period (in the initial 6 months) pre scores Mean±SD:  5.8±2.5 (n=65)  Intervention period (later 6 months) pre- scores Mean±SD: 5.7±2.6 (n= 40) | Control period post value given: 5.7±2.6 (n= 65)  Intervention period post value: 5.5±2.7 (n=40)  (intervention pre= control post) |
| Wolever RQ et al, 2010 [53] | Primary | Self- report  Tool:   - Morisky Adherence Scale [scored: 0-9] - Patients also answered the adherence question: ‘have you missed a medication dose in the past week?’ | Adherence measure scores  and,  No. of patients | Morisky scores:  Mean ± SD  ***I***: 6.7±0.96  ***C***: 6.7±1.25  No. of patients who reported to have missed dose in past 7 days:  ***I***: 51.9%  ***C***: not given | Morisky scores  (Mean ± SD):  ***I***: 7.2±0.97*  ***C***: 6.9±1.25  Number of patients who reported to have missed dose in past 7 days:  ***I***: 7.4%*  ***C***: not given |
| Zhang Y et al, 2010 [54] | Primary | Pharmacy and medical claims  Tool:  Medication possession ratio (MPR)   - Calculated as the proportion of days during a given year a subject had possession of drugs used to treat the condition under evaluation   Treatment intensity:   - Calculated as the average number of pills per day during which a patient was receiving medication treatment for the disease in a year.   Used in analysis:   - Good medication adherence:   MPR ≥ 0.80 (Or 80, i.e. MPR*100)   - **Likelihood of good medication** adherence expressed as proportion of patients with MPR≥0.8 - **Treatment intensity** | Adherence scores | MPR (in %) :  ***I***:  No coverage = 57  $150 cap= 77.3  $ 350 cap= 75.4  Comparison group:  No cap: 81.8  Likelihood of good medication adherence::  ***I***:  No coverage = 39.7  $150 cap= 68.0  $ 350 cap= 62  Comparison group:  No cap: 70.6  Treatment intensity :  ***I***:  No coverage = 0.98  $150 cap= 1.12  $ 350 cap= 1.11  Comparison group:  No cap: 1.29 | MPR (in %):  ***I***:  No coverage = 69.6  $150 cap= 76.2  $ 350 cap= 73.3  Comparison group:  No cap: 78.2  Likelihood of good medication adherence:  ***I:***  No coverage = 57.2  $150 cap= 67.1  $ 350 cap= 61.9  Comparison group:  No cap: 66.6  Treatment intensity* :  ***I:***  No coverage = 1.16  $150 cap= 1.26  $ 350 cap= 1.18  Comparison group:  No cap: 1.34 |
| Gracia- Huidobro D et al, 2011 [55] | Secondary | Self- report  Tool: Morisky–Green Scale (validated in Spanish) [Scored : 1- 4] | Adherence measure score | Mean (±SD):  ***I***: 2.4(±0.8)  ***C1***: 2.4(±0.8)  ***C2***: 2.4(±0.9) | Mean (±SD):  @ 12 month:  ***I***: 2.4(±1.1)  ***C1***: 2.6(±1.1)  ***C2****: 2.7(±1.2) |
| Khan MA et al, 2011 [56] | Primary | Self- report  Tool:  1.Morisky scale (4- item)  *[Scored: 0- 4] (lower the score, better*  *the adherence)*  2. SDSCA – (1- item)  *[Scored: 0- 7]* | Adherence measure score | Morisky mean(SD):  ***C:*** 1.2 (1.2);  ***I:*** 1.0 (1.2)  SDSCA, mean(SD):  ***C***: 5.6 (2.3)  ***I:*** 5.0 (2.7) | Morisky mean(SD):  ***C***: 0.8 (1.0);  ***I:*** 0.8 (1.1)  SDSCA, mean(SD):  ***C***: 6.2 (1.8)  ***I:*** 5.0 (2.9) |
| Mehuys E et al, 2011 [57] | Secondary | Pharmacy refill claims & Self report  Tools:  Pharmacy refill:   - Prescription refill rate & Self report - Calculation done as: Adherence Rate (%) = ‘total days supplied during the study - days of last supply in the study’ divided by ‘last claim date in the study - first claim date in the study’ multiplied by 100   Self-report:   - Assessed at the end of the study by asking patients: *How often do you not take your OHA as prescribed? with permitted answers of (i) never, (ii) 1–2 times ⁄ year, (iii) 1–2 times ⁄ month, (iv) 1–2 times ⁄ week, or (v) daily.* | No. of patient | Judged from prescription refill rate, adherence was very high, for both groups. The group median was: ***C:***94.7% and***I:***99.7%  (*some had rate calculated even up to 200%)*🡪 data not used for further analysis therefore  Self –report:  ***C*** (% patient):  Never: 64.2  1- 2 times/ yr: 14.2  1- 2 times/ mth: 12.7  1- 2 times/ wk: 7.5  Daily : 1.5  ***I*** (% patient):  Never: 59.9  1- 2 times/ yr: 19.1  1- 2 times/ mth: 10.9  1- 2 times/ wk: 8.8  Daily : 1.4 | Not assessed with prescription refill  Self- report:  ***C*** (% patient):  Never: 61  1- 2 times/ yr: 15.5  1- 2 times/ month: 15.5  1- 2 times/ week: 7.3  Daily : 0.8  ***I*** (% patient):Never(% patient): 61.9  1- 2 times/ yr: 19.7  1- 2 times/ month: 10.2  1- 2 times/ week: 6.8  Daily : 1.4 |
| Mitchell B et al, 2011 [58] | Primary | Self- report  Tool: Brief Medication Questionnaire (BMQ)   - 3 screens: Regimen screen (range 0- 8); Belief screen (range 0- 2); Recall screen (range 0- 2) - The regimen screen in the BMQ measures adherence behaviours in the past 7 days; Regimen Screen Scale: 0- 8 🡪 lower score the better adherence | Adherence measure score | Regimen screen:  1.2 (1.08- 1.34)  Belief screen:  0.62 (0.54- 0.74)  Recall screen:  0.98 (0.92- 1.04) | Regimen screen* :  0.84 (0.73- 0.95)  Belief screen* :  0.37 (0.3- 0.44)  Recall screen:  1.00 (0.93- 1.06) [n= 347] |
| Piette JD et al, 2011 [59] | Secondary | Self- report  Tool: Morisky Medication Adherence Scale  [Scored: 0-9] | Adherence measure score | Mean ± SD:  ***C***: 8.5±1.0***I***: : 8.4±0.7 | Mean ± SD:  ***C***: 8.6±1.0  ***I:*** 8.6±1.0 |
| Ramanath KV, Santhosh YL, 2011 [60] | Secondary | Self- report  Tool: MMAS [4 item scale, Score 0- 4,  higher the better] | Adherence measure score | Approx. values as the figures are projected from the graph  ***I***: 1.75  ***C***: 1.8 | Approximate values as the figures are projected from the graph  @Follow up:  1^st^:  ***I***:2.15; ***C***: 2.1  2^nd^*:  ***I***: 2.6;***C***: 2.3  3^rd^*:  ***I***: 2.9; ***C***: 2.3  *'Significant improvement was observed in all follow ups in intervention group but variation in control group and in the 2^nd^ and final follow, the change was strongly significant'* |
| Shetty AS et al, 2011 [61] | Primary | Self- report  Tool:   - *“ a validated questionnaire*” - "*patients were requested to keep a diary to note deviations in drug / wk*" | Can't be certain | Data not presented  States "*drug prescriptions were followed satisfactorily by both groups*" | Data not presented |
| Smith SM et al, 2011 [62] | Secondary | Self -report  Tool: MARS   - [Score range:: 5- 25, higher score the better adherence] | Adherence measure score | Mean(SD):  ***C***:23.8(1.8)  ***I***:23.9(1.7) | Mean(SD):  ***C***:23.9(0.4)  ***I***:24.1(0.3) |
| Wakefield BJ et al, 2011 [63] | Secondary | Self- report  Tool:   - *'adherence assessed using validated regimen adherence scale, which addresses medication, diet, exercise, and BG testing*' - *The tool developed as a doctoral dissertation🡪 the scale was validated in a sample of 181, type1 patients; had moderate reliability (alpha = 0.73) score, and item-total correlations ranged from 0.34 to 0.60.* | Can't be certain | Not given | Not shown  Says *'adherence improved overtime for all groups but there were no significant difference among the groups*' |
| Walker EA et al, 2011 [64] | Secondary | Self- report  Tools:  1. *MPR (Medication possession ratio)*  *Calculated using pharmacy claims (i.e.*  *administrative data)*  2. 4- item Morisky Scale  Scores ≤2 in Morisky scale analysis was  considered non adherent  3. SDSCA questionnaire (1- item):   - *‘how many days in the most recent week were diabetes pills taken as prescribed?” (score 0- 7)*   Considered for the statistical analysis:  Change in Medication Possession Ratio (ΔMPR ≥ 20%), calculated as follow up minus baseline | Number of patients reporting adherence | Patients (%) reporting adherence:  Morisky Scale ≤ 2(%):  **CC1**: 35.1%  ***CC2***: 38.6%  Patient (%) reporting taking diabetes pills <7 days/wk (SDSCA):  ***CC1***: 27.9%  ***CC2***: 25.4% | Morisky data and SDSCA data are not presented in the text post intervention, ΔMPR≥ 20% is discussed, with the following major finding:   - Among those not using insulin, there was a significant (p= 0.001) linear trend with ∆MPR ≥ 20%, for the number of interventions call received. - The telephone intervention was not associated with a change in medication adherence if the regimen included insulin |
| Barron JJ et al, 2012 [65] | Primary | Medical and pharmacy claims  (retrospective analysis of medical and pharmacy claims data)  Tool: Medication Possession Ratio (MPR)   - The sum of the total supply (number of days) of any oral anti- diabetes medication divided by a 1- year period (the duration of the follow up). - Patients with an MPR score ≥ 0.8 were defined as adherent | Adherence score measure  and  proportions (%) of patients who were adherent | Baseline values:  Study 1 (PA)  MPR [mean(SD)]  ***I***: 0.64(0.27)  ***C***: 0.59 (0.26)*  Study 2 (TDES):  ***I***: 0.76 (0.22)  ***C***: 0.68 (0.28)* | @1- year follow up:  MPR (estimated mean)  Study 1(PA):  ***I***: 0.68; ***C***: 0.64  Study 2(TDES):  ***I***: 0.82; ***C***: 0.77  Proportions of patient adherent to diabetic medication at 1- year follow up:  Study 1(PA):  ***I***: 43% *; ***C***: 36%  Study 2 (TDES):  ***I***: 68%; ***C***: 65% |
| Bogner HR et al, 2012 [66] | Primary | Electronic measures  Tool: MEMS | No. of patient (≥80% adherent) | Patient population who are ≥80% adherent to OHA:  ***I***: 35.9%; ***C***: 42% | Patient population who are ≥80% adherent to OHA:  (approx. values as projected from graph)  @6 weeks*:  ***I***: 62%; ***C***: 33%  @12 weeks*:  ***I***: 64%; ***C***: 31% |
| Brennan TA et al, 2012 [67] | Primary | Pharmacy claims  Tool: Medication possession ratio  ‘Monthly use of medications to treat diabetes and the change in monthly use’ is used to analyse adherence | Adherence measure score (change in monthly supply) | No data pre- intervention  (only change in monthly supply of OHAs have been reported) | During program% change on monthly supply of OHAs:  Full sample*: 2.1  Retail group*: 3.9  Pharmacy benefit management program*: 1.7  After program % change on monthly supply of OHAs:  Full sample: 1.0  Retail group: 1.2  Pharmacy benefit management group: 1.0 |
| Choi SE, Rush EB, 2012 [68] | Primary | Self- report  Tool: SDSCA (1- item)   - [scored 0- 7] | Adherence measure score | Mean(SD):  6.37(1.88) | Mean(SD)  Post- program: 6.37 (1.92)  &  Follow-up: 6.61 (1.54) |
| Farmer A et al, 2012 [69] | Primary | Electronic measure & Self- report  Tool:  Electronic measures: MEMS  Self- report:   - MARS, a 5 – item self-report scale each with item responses scored on a five point scale (scored 5- 25); - % of days over a 12- wk period on which the correct number of doses was taken each day | Adherence measure score | Baseline measured by MEMS is not presented  MARS Score(SD):  ***I***.: 23.6 (2.3) ***CC***: 23.6 (2.8) | MEMS:  Reported as days correct dose taken (SD)*%  ***I:*** 77.4 (26.3)  ***CC***: 69.0 (30.8)  MARS Score(SD):  ***I***: 23.6 (2.6)  ***CC***: 24.1 (1.6) |
| Kroese FM et al, 2012 [70] | Secondary | Self- report  Tool: Medication Adherence Report Scale (MARS)   - The degree to which the patients did not take their medications as prescribed; Scored 1(always true) to 5 (never true) | Adherence score measure | Scores(SD):  @ T1:  4.65 (0.57) | Scores (SD):  @ T2 (n= 86):  4.79 (0.32)  and,  @ T3 (n= 53):  how group: 4.83(0.30)  why group: 4.76(0.30) |
| Mellitus Janice C-M et al, 2012 [71] | Primary | Self- report  Tool: Self- Care Practices Measurement questionnaire (DSCPM)   - based on the patients’ response to *‘how frequently they engaged in medication taking practices*’ using five responses from never (0%) to all of the time (100%) | No. of patients with adherence score ‘50- 100%’ & ‘0- 25%’ | No. of patients with baseline adherence scores:  0- 25% : 0, &  50- 100%: 9 | No. of patients with post intervention adherence scores*:  0- 25% : 0  50- 100%: 10 |
| Odegard PS, Christensen DB, 2012 [72] | Primary | Pharmacy/ prescription refills  Tool:  Medication possession ratio (MPR)  Refill gap ‘max. days during 12 month per-period’ | Adherence score;  Refill gap(no. of days) and  Number of patients (with MPR>0.8) | MPR during 12 month pre-period:  ***I***: 0.86  ***C***:0.84  Refill gap ‘max. days during 12 month pre-period’:  ***C***: 22.7; ***I***.: 22.3  MPR>0.8 during 12 month pre-period  ***C***: 65.2%; ***I***.: 74.4 %  (p= 0.029) | For the study group,  @6- months  MPR-improved from 0.90 to 0.92 (p= 0.16)  and  @12 months:  MPR* improved from 0.85 to 0.90 (p<0.01)  MPR remained nearly unchanged for control participants and was slightly worse at 12 months |
| Ramanath KV et al, 2012 [73] | Primary | Self- report  Tool: Morisky Medication Adherence Scale (MMAS)- 4- item  [scored out of 4, higher the score, better the adherence] | Adherence measure score | Mean ± SD  ***I***: 1.75±1.29  ***C***: 3.04±1.00 | Mean ± SD  ***I***:  1^st^ follow up: 3.08±0.72  2^nd^ follow up: 3.67±0.56*  ***C***:  1^st^ follow up: 3.08±0.93  2^nd^ follow up: 3.13±0.74 |
| Vervloet M et al, 2012 [74] | Primary | Electronic measure  Tool: Real Time Medication Monitoring (RTMM) system  Measured using 3 different measures:  1. No. of days without dosing, calculated by adding up the days on which no single dose was taken;  2. Proportion of missed dose, calculated as the number of dispenser openings subtracted from the number of prescribed daily dose;  and  3. Proportion of doses taken within agreed and predefined standardized time windows, calculated by dividing the total number of dispenser openings within the time window (predefined) by the total number of prescribed doses during the intervention period | Different aspects of medicine taking behaviour | [Calculations have been done over the length of the intervention period and compared between the control and intervention group, as shown in the next column | Over the intervention period:  Days without dosing:  ***I:***11.9±18.8; ***C***: 13.8±14.5  Doses taken within agreed time period*:  ***I:***56.7±23.8; ***C:*** 43.2±26.2  Missed doses (%):  ***I:***14.5±15.7; ***C:***19.2±16.0  Doses taken within std window*:  1hour:  ***I:***50.3±22.8; ***C***: 38.7±23.0  2hour:  ***I:***71.4±23.8; ***C***:57.2±23.4  3hour:  ***I*:**77.3±21.6; ***C***: 65.3±22.6  4hour:  ***I:***80.5±20.5; ***C:*** 70.2±21.6 |
| Zolfaghari M et al, 2012 [75] | Secondary | Self –report  Tool:  Self- care diabetes questionnaire, consisting of 11 items relating to diabetes medication taking along with other questions in areas of diet and exercise  *(questionnaire was verified by instructors of Teheran University, Chronbach α was 0.87*)   - Scoring: scored 4 for achieved goal and 0 for a non- achieved goal and calculated as percentage | Adherence measure score | Mean ± SD (%) value for adherence @ baseline:  ***CC1***: :  75.48 ± 14.33  and  ***CC2***:  73.27± 14.75 | Mean ± SD (%)value for adherence @ 3 months (post intervention):  ***CC1:***  91.13±11.61  and  ***CC2:***  94.73±7.63 |

***** statistically significant outcome (p<0.05)
