# Supplementary material for: A Systematic Review of Interventions Addressing Adherence to Anti-Diabetic Medications in Patients with Type 2 Diabetes—Impact on Adherence
Source: PLoS One. 2015 Feb 24;10(2):e0118296. doi: 10.1371/journal.pone.0118296 (PMC4339210; doi:10.1371/journal.pone.0118296)
Supplement: S1 Fig — S1_Figure.docx (DOCX) [file pone.0118296.s006.docx]

**Figure S1: Literature review search strategy**

|  | Group of keywords used |
| --- | --- |
| 1 | adherence, patient adherence, medication adherence, therapy adherence, treatment adherence, medication intake adherence, medication compliance, patient compliance, persistence, concordance  drug therapy, initiation, execution, cessation, medication therapy management, patient care/ patient centered care |
| 2 | diabetes mellitus, type 2 diabetes mellitus, non- insulin dependent diabetes  anti-diabetic agent, hypoglyce*mic agent, hypoglyce*mic |
| 3 | Intervention study, develop intervention, evaluate intervention, implement intervention, intervention model, complex intervention, intervention assessment  Behavio*r/ behavio*r therapy, patient education as topic |
|  | |

Concept

**3. Intervention**

**2. Diabetes, type 2 and/ or anti-diabetic drugs**

**1. Adherence**

Category 1:

Issue Adherence adherence, patient adherence, medication adherence, therapy adherence, treatment adherence, medication intake adherence, medication compliance, patient compliance, persistence, concordance

drug therapy, initiation, execution, cessation, medication therapy management, patient care/ patient centered care

Category 2:

Disease/ Drugs Diabetes diabetes mellitus, type 2 diabetes mellitus, non- insulin dependent diabetes

Antidiabetic agents antidaibetic agent, hypoglyce*mic agent, hypoglyce*mic

Category 3:

Focus Intervention Intervention study, develop intervention, evaluate intervention, implement intervention, intervention model, complex intervention, intervention assessment

behaviour/ behavior therapy, patient education as topic

LIMIT Human

Year: 2000 to 2013/ current, English
