# Supplementary material for: A Systematic Review of Interventions Addressing Adherence to Anti-Diabetic Medications in Patients with Type 2 Diabetes—Impact on Adherence
Source: PLoS One. 2015 Feb 24;10(2):e0118296. doi: 10.1371/journal.pone.0118296 (PMC4339210; doi:10.1371/journal.pone.0118296)
Supplement: S2 Fig — S2_Figure.docx (DOCX) [file pone.0118296.s007.docx]

**Figure S2: Citation selection flowchart for the review**

6662 citations were identified from searches in multiple electronic databases

181 did not meet the inclusion criteria

- 28: review articles on various issues relating to diabetes, management interventions or related
- 20: articles that detailed the method of intervention only
- 95: did not assess adherence to medications
- 11: also dealt with comorbid conditions/ medications without separately analyzing for type 2 cases and/ or anti-diabetic medications
- 17: included both type 1 & type 2 patients or diabetes in general without separate analysis for type 2 cases
- 10: other reasons (eg no intervention, adherence not measured pre and post)

6416 citations were removed

- 1177 duplicates
- 251 dealt exclusively with Type 1 diabetes and/or diabetes in children and/ or adolescents (removed based on title)
- 4988 were either not ‘research’ articles (eg. handbook, letters, short-communication), or they did not meet inclusion criteria (removed based on title and/ or abstract)

3 articles obtained from hand search

52 articles were included in the review

49 articles met the inclusion criteria

230 articles were reviewed in full text to assess for inclusion

246 citations were considered for full text review

16 citations could not be reviewed in full text

- 12:conference/ Posters/ Meeting abstracts
- 4: abstract in English but full text in other languages
