# Supplementary material for: A Systematic Review of Interventions Addressing Adherence to Anti-Diabetic Medications in Patients with Type 2 Diabetes—Impact on Adherence
Source: PLoS One. 2015 Feb 24;10(2):e0118296. doi: 10.1371/journal.pone.0118296 (PMC4339210; doi:10.1371/journal.pone.0118296)
Supplement: S3 Fig — S3_Figure.doc (DOC) [file pone.0118296.s008.doc]

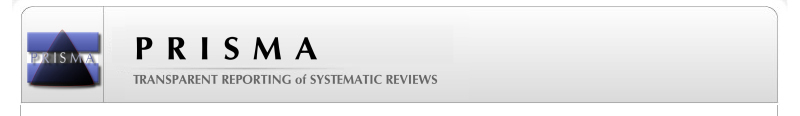
**Figure S3: PRISMA 2009 Flow Diagram**

**Screening**

**Included**

**Eligibility**

**Identification**

Records identified through database searching
(n =6662)

Additional records identified through other sources
(n =0)

Records after duplicates removed
(n = 5485)

Records screened
(n = 246)

Records excluded
(n = 5239)

Full-text articles assessed for eligibility
(n = 230)

Full-text articles excluded, with reasons
(n = 181)

Reasons:

28: review articles on issues relating to diabetes, management interventions or related (not matching inclusion criteria)

20: articles that detailed the method of intervention only

95: did not assess adherence to medications

11: also dealt with comorbid conditions/ medications without separately analyzing for type 2 cases and/ or anti-diabetic medications

17: included both type 1 & type 2 patients or diabetes in general without separate analysis for type 2 cases

10: other reasons (eg no intervention, adherence not measured pre and post)

Studies included in qualitative synthesis
(n =49)

Total studies included in the review
(n = 52)

Additional records identified through hand search of retrieved articles (n=3)
