# Supplementary material for: A Systematic Review of Interventions Addressing Adherence to Anti-Diabetic Medications in Patients with Type 2 Diabetes—Impact on Adherence
Source: PLoS One. 2015 Feb 24;10(2):e0118296. doi: 10.1371/journal.pone.0118296 (PMC4339210; doi:10.1371/journal.pone.0118296)
Supplement: S1 Appendix — S1_Appendix.docx (DOCX) [file pone.0118296.s009.docx]

**Appendix S1**

**Full electronic search strategy for Medline database**

Database: Ovid MEDLINE(R) <1946 to March Week 4 2013>

1 exp Medication Adherence/ or adherence.mp. (74618)
2 patient compliance.mp. or exp Patient Compliance/ (52925)
3 persistence.mp. (52272)
4 concordance.mp. (22663)
5 medication therapy management.mp. or exp Medication Therapy Management/ or exp Pharmacy/ (20019)
6 treatment regimen.mp. (7857)
7 1 or 2 or 3 or 4 or 5 or 6 (212841)
8 hypoglycemic agent.mp. or exp Hypoglycemic Agents/ (183540)
9 antidiabetics.mp. (2287)
10 8 or 9 (183747)
11 type 2 diabetes mellitus.mp. or exp Diabetes Mellitus, Type 2/ (80197)
12 7 and 10 (2494)
13 10 or 11 (238940)
14 12 and 13 (2494)
15 intervention study.mp. or exp Intervention Studies/ (10073)
16 exp Patient Education as Topic/ or complex intervention.mp. (66959)
17 evaluate intervention.mp. (89)
18 exp Behavior Therapy/ or implement intervention.mp. or exp Health Promotion/ (97498)
19 develop intervention.mp. or exp Health Promotion/ (49751)
20 15 or 16 or 17 or 18 or 19 (168838)
21 7 and 13 and 20 (565)
22 limit 21 to (english language and humans and yr="2000 -Current") (390)
