# Supplementary material for: A Systematic Review of Interventions Addressing Adherence to Anti-Diabetic Medications in Patients with Type 2 Diabetes—Impact on Adherence
Source: PLoS One. 2015 Feb 24;10(2):e0118296. doi: 10.1371/journal.pone.0118296 (PMC4339210; doi:10.1371/journal.pone.0118296)
Supplement: S2 Appendix — S2_Appendix.docx (DOCX) [file pone.0118296.s010.docx]

**Appendix S2: Intervention Summary and Impact**

| Study | Brief description of intervention(s), control and/or comparison activities and reported impact |
| --- | --- |
| Hendricks LE, Hendricks RT, 2000 [24] | Intervention: A series of 2- hour diabetes self- management skills training classes, and telephone follow-ups were provided. Two groups of intervention patients were included; one group received monthly telephone follow up for 6 months ***(CC1)***, while the other received the follow up at 3- months’ interval over a 6-month period ***(CC2).*** The telephone follow-up was 10-15 minutes duration (each), conducted to evaluate: patients’ progress in achieving their treatment goals; determine whether they were having any problems in self- managing their diabetes; and track the progress of selected outcomes. Any concerns, knowledge deficits regarding diet, exercise, medication or blood glucose monitoring were addressed by the investigator. During the 6- month follow up period, participants who received monthly follow-up were contacted an average of 3.7 times (SD= 2). Those who received follow up at 3-monthly intervals were contacted an average of 2 times (SD= 0.76)  **Impact**: Nothing significant |
| Grant RW et al, 2003 [25] | Intervention ***(I)***: Initial phone interview to assess barriers &overall adherence, with detailed assessment on medication use [based on questionnaire] conducted by pharmacists; followed by medicine-specific patient education and arrangements for social service or nutritional consultation as required. An email, summarising medication discrepancies (identified by comparison of patients’ medication with updated electronic medical record), adherence barriers and offer for help to arrange follow up appointment was also delivered to patients’ primary care provider. Follow up was conducted after 3 months.  Control ***(C)***: Did not receive any intervention. Pre and post assessment of adherence barriers and overall adherence was conducted at 3 months.  **Impact**: 30 out of 45 medication discrepancies were resolved; and 6 medications in regimen was changed; adherence to exercise improved. |
| Kim HS, Oh JA, 2003 [26] | Intervention ***(I)***: Diabetes care booklet and a daily log, containing information about the nature of the disease, risk factors, diet, exercise, drug therapy, hypoglycaemia/ hyperglycaemia management, and how to record a daily log, were provided to the participants before the intervention and were asked to log blood glucose levels, daily diet and exercise records.  The intervention consisted of continuing education and reinforcement of diet, exercise, medication adjustment recommendations, and self-monitoring of blood glucose levels. The education was provided via telephone. Patients were contacted at least twice a week for the first month and then weekly for the second and third months (16 calls/ patient on average, average time: 25 minutes/ call). A registered dietician analysed participants’ daily food intake and made recommendations for appropriate diabetic dietary control. The results and recommendations were mailed to the participants. Medications were also adjusted after reviewing the blood glucose log and discussing glucose values with the patients, and the changes were communicated to the patients’ doctors.  Control ***(C)***: Routine care (visit to a physician every 3 months).  **Impact**: Improvements in HbA1c; and greater adherence to diet and blood glucose testing in the intervention group post-test. |
| Maddigan SL et al, 2004 [27] | Two rural health regions in close proximity were randomly allocated as a ‘control’ and ‘intervention’ region. Both regions received 3 bimonthly visits by the Canadian Diabetes Association Travelling Diabetes Resource Program (CDA- TDRP).  Intervention ***(I)***: The intervention region received a ‘*diabetes outreach service*’, consisting of a team of specialist physicians, nurse educators, dieticians and pharmacists, who travelled to the larger communities in that region, every month for 6 months, to deliver targeted educational messages, focused on the interaction of blood pressure, cholesterol, and glucose on macro-vascular complications of diabetes). Educational messages were delivered, by specialist physicians to small groups of 2 to 6 primary care physicians; using ‘*group academic detailing’* technique. The intervention had different components targeted to different sets of health care providers. For example, the primary care physicians of the region participated in small group discussions of real and theoretical cases related to risk factors (led by specialists), one- on- one academic detailing by a trained pharmacist, and a referral service for a limited number of patients. The allied health care professionals ‘in- services’ focused on the importance and management of risk factors and were provided in groups of 8 to 30.  Control ***(C)***: In the control region, the local providers received only 3 bimonthly visits by the CDA- TDRP.  **Impact**: Health related quality of life, satisfaction with diabetes care and general medical care, and scores in Diabetes Lifestyle Form (DLF) significantly improved in the intervention group compared to control |
| Rosen MI et al, 2004 [28] | Intervention ***(I)***: Patients were “cue dose” trained and were given *SmartCaps* (MEMS caps that displayed the number of hours since the last bottle opening, which could be programmed to beep at predetermined times). They were instructed to consider cues that would help them remember to take their medication. The MEMS data were given to providers each month as it was collected. The providers were contacted and suggested to discuss MEMS data with their patients during the scheduled appointments. The intervention phase lasted for 16 weeks.  Control ***(C)***: Out of the 17 patients, the first five patients assigned to the control, received supportive counselling based on self-reported data without presentation of MEMS; and the next 12 patients received assessments only with no active counselling and no presentation of MEMS data to providers.  **Impact**: “Cue dose” training was associated with significantly better adherence to metformin |
| Schectman JM et al, 2004 [29] | 83 resident and faculty physicians were provided with a 2-page report on each of their diabetic patients. Page 1 contained latest information on metabolic control, renal function, lipid values, and ophthalmology encounters, and page 2 contained summary refill-based adherence data for diabetes medications, including percentage adherence for oral agents and average daily dose of anti- diabetic medications. The data were calculated based on the dispensed amount over the refill interval from the pharmacy.  Intervention ***(I)***: The physicians were invited to a 30- minute educational session, conducted in 5 small sessions in order to review feedback reports, techniques for patient adherence assessment, barrier evaluation, and intervention strategies; physicians attending 1 of 5 small group sessions were allocated to the intervention group.  Comparison ***(CC)***: Comparison group consisted of physicians who received the 2-page report but did not take part in the educational session.  **Impact**: A significantly greater improvement on patient refill adherence for physicians attending the educational session; the change in refill adherence of patients attending the study physician to anti-hypertensive medications (for which the physicians did not get the feedback) was also highly significant in the physician group who received feedback and education session, and not significant in the comparison group. |
| Wermeille J et al, 2004 [30] | Intervention: With the help of research pharmacist, the community pharmacist did a review, based on national guidelines, for each patient to identify any drug related problems. The information required was obtained from the general practice notes, the community pharmacy Patient Medication Record system and a structured patient interview (approx. 30 min). The patient’s information was compiled in a specially designed patient medication profile, and a pharmaceutical care plan (PCP) was generated. For each pharmaceutical care issue (PCI) identified, a desired output and proposed action were documented. All PCPs were discussed with a second community pharmacist and two academic pharmacists, including the research pharmacist. The PCP was then discussed face-to-face with the patient’s general practitioner (GP). Where PCPs involved several PCIs, prioritization was placed on optimising diabetes, hypertension and hyper-lipidaemia drug therapy. All agreed actions were documented and a copy retained by the GP.A second patient interview was conducted 24 to 28 weeks from the initial interview, to establish if identified PCIs had been resolved and to assess outcome measures.  **Impact**: Significant reduction in the biologic measures, such as, HbA1c, blood pressure, total cholesterol; improvement in patient knowledge for oral hypoglycaemic therapy |
| Odegard PS et al, 2005 [31] | Intervention ***(I)***: The intervention consisted of formulation of diabetes care plan (DCP) in collaboration with patient and provider by the pharmacist, and its implementation. The intervention was started one week after the baseline interview with the patient to develop the DCP. The pharmacist then maintained a regular contact with the patient on a weekly basis either by telephone or in- person, in order to follow up on the DCP, modify if needed and provide necessary referrals. The progress as per DCP was assessed on these meetings. Once it was determined that the care needs were progressing as outlined, follow-up phone call frequency was reduced to monthly through the 6-month intervention period.  Control ***(C)***: Patients were not provided with 'diabetes education' during the baseline interview, and were instructed to continue normal care.  **Impact**: Decrease in HbA1c in the intervention group but not significantly different compared to the control group; Medication appropriateness index scores for intervention subjects appeared improved by 50% at 6 months (not significant). |
| Keeratiyutawong P et al, 2006 [32] | Intervention ***(I)***: The intervention consisted of 5 hours of cognitive improvement sessions and 5 hours for skills training, conducted in5 sessions that covered the following: (1) pathology of diabetes mellitus, cognitive restructuring and goal setting skills; (2) dietary control and communication skills; (3)diabetes medication, and problem solving skills; (4) foot care and self-monitoring; and (5) exercise. Session 1 was conducted on Saturday and Sunday from 9.00 to 11.00 a.m., followed by the other sessions (2 hours/ week) conducted in the following week. Participants were also provided with a set of written diabetes material and 5 videotapes about diabetes self-care. A follow-up telephone call was conducted at the 3^rd^ and the 5^th^month to discuss the participants’ diabetes self-management and any associated problems. Support and reinforcement were provided to encourage the participants to continue with their self-management practices.  Control ***(C)***: Participants received a set of written diabetes materials at baseline. They also attended diabetes education (1- hour) class at a diabetes clinic on the day of the physician’s visit and watched 5 videotapes about diabetes care (one or two videotapes per visit), conducted by the diabetes health care team of the hospital. The participants were kept in contact via telephone calls at 1, 3 and 5 months to remind them of appointments dates and to report laboratory results. Data were collected at 3 and 6 months.  **Impact**: Significant difference in knowledge, diabetes self-care activities (DSCA) and diabetes quality of life mean scores in at least one of the three time points in intervention group. |
| Kim HS et al, 2006 [33] | Intervention: Patients were asked to input their blood glucose levels every day by cellular phone or the internet; and to input their medicines for diabetes. The researcher had access to information provided by each patient, their personal history and other patient information, for eg medications and baseline laboratory data. Based on the overall information, weekly optimal recommendations were sent to each patient by SMS. The intervention consisted of continuous education and reinforcement of diet, exercise, medication adjustment, and frequent self-monitoring of blood glucose levels, via SMS, based on a protocol approved by the physician after reviewing the blood glucose log and discussing glucose values with patients. All medication adjustments were communicated to the subjects’ physicians. Patients were withdrawn from the study group if they did not report the blood glucose levels for more than 4 consecutive weeks despite the warnings sent (sent if the patient did not forward a blood glucose level for more than 1 week).  **Impact**:HbA1c, adherence to medication, 30- minute physical exercise and foot care improved significantly |
| Vincent D et al, 2007 [34] | Intervention ***(I)***: The intervention consisted of educational content, cooking demonstrations, and group support sessions (2 hours every week for 8 weeks). The content included pathophysiology of diabetes, complications, treatment modalities, diet, exercise, and self-management strategies, self-monitoring of blood glucose. The didactic sessions included stress management, heredity and cultural issues; participants were encouraged to bring a support person to the sessions. Strategies to promote self-efficacy were incorporated and included skill mastery of self-glucose monitoring, problem solving, and verbal persuasion.  Control ***(C)***: The control group received usual care and education given at the clinic, which consisted of a 10- to 15-min encounter with a physician or nurse practitioner 2 to 4 times per year.  **Impact**: A positive clinical and statistical effect on diabetes knowledge, weight and body mass index. Improvements were also noted in self-efficacy scores, blood glucose, and HbA1C (but did not reach statistical significance). |
| Faridi Z et al,  2008 [35] | Intervention ***(I)***: Patients attended a 1-day training workshop, to learn how to use NICHE technology (“*an interactive informational feedback system that uses wireless remote technology to provide tailored feedback and reminders, based on patient-specific data, to patients and providers via messages on cellular phones”)*for T2D management. After training, patients participated in a 3-month intervention, during which they were required to test their glucose once daily (upon awakening), wear their pedometers during the day, and upload data onto the NICHE server once daily. Both patients and providers were able to view readings from uploaded data. Based on the uploaded data, each patient received tailored messages via mobile phone.  Control ***(C)***: Control subjects continued with standard diabetes self-management and tracked their step count using a pedometer.  **Impact**: Improvement in HbA1c (not significant); significant improvement in the self- efficacy scores in the intervention group. |
| Quinn CC et al, 2008 [36] | Intervention ***(I)***: Participants received a Bluetooth-enabled One Touch Ultra Blood Glucose (BG) meter, BG testing strips and lancets, along with a Nokia cell phone that was equipped with ‘WellDoc’s proprietary Diabetes Manager software’. Patients’ logbooks were sent electronically to their HCPs by the WellDoc^TM^ System (WDS) every 4 weeks, or sooner, along with the analyses of data, trends, and patient behaviour. WDS was designed to serve as a virtual coach for patients and virtual endocrinologist for the health care professional (HCP).The system communicated suggested medication changes to patients, and all suggested changes to patients’ therapy regimes to the HCP; the choice whether to put into practice the suggestion lied with the HCP. Feedback to the patient was provided on nutrition, lifestyle, stage of change, and self-management skills. If it was felt necessary that that the patient required a live session for nutrition or self-management help, the HCP was notified, and the patient was given a referral to a Certified Diabetes Educator and/or nutritionist within the patient’s healthcare network.  Control ***(C)***: Patients were provided with One Touch Ultra™ BG meters, BG testing strips and lancets, and were asked to fax or call in their BG logbooks every 2 weeks to their HCPs until their BG levels were stabilized in the target ranges or until their HCPs changed testing frequency. The treating HCPs were asked to follow their usual standard of care for the patients’ diabetes management.  **Impact:** Significant improvement in HbA1c; medication intensification and medication errors identified were significant post intervention in the intervention group; providers’ diabetes management improved significantly by receipt of blood sugar data; patients self-management skills improved; physicians received data from significantly more intervention patients; medication adherence was better in intervention group (not significant) post intervention. HCP and patient satisfaction with the system was statistically significantly better in the intervention group. |
| Utz SW et al, 2008 [37] | Study consisted of comparison between group delivered and individual diabetes self-management (DSM) interventions.  Group Diabetes Self- Management ***(CC1)***: Culturally tailored DSM Education was delivered by diabetes educators for 8 weeks (2h/ wk) in groups. The features of the sessions were: learning in a supportive atmosphere; story-telling related to diabetes care and overcoming chronic illness, using figurative language typical of the region, delivered by an African American woman as a “lesson” to begin most sessions; simple colourful 1- page education materials provided as handouts; “lessons” based on 7 areas of diabetes self-management (based on ADA) delivered using an interactive approach that emphasised respect and empowerment, and incorporated activities and problem solving techniques. African American group leaders and role models were involved to emphasize adult learning principles, problem-solving about diabetes management issues; guiding hands-on activities; offering ideas about ways to decrease costs of care and guidelines for using complementary and/or alternative therapies; involving supportive family and/or friends in selected sessions; and celebrating successes, including giving a completion certificate at the end of the program.  Individual Diabetes Self-Management ***(CC2)***: Individuals met a Diabetes Educator 3 times and received the following intervention: **week 1**—setting a goal (based on 7 areas of self- care) and receiving the notebook of educational materials; **week 4**—reviewing progress towards the goal, problem-solving strategies to improve self-care, offering additional information or resources, and receiving a cook book, exercise videotape, pedometer, and/or foot care kit (based on the goal set and as preferred); **week 8**—final review of progress towards individual goal, offering information and resources, discussion about diabetes self-management achieved, and plans for future self-care.'  **Impact**: Group diabetes self-management had a better score (but non- significant) in activities like carbohydrate spacing, comprehensive foot care items measures, measures of self- efficacy, high rates of goal achievement |
| Babamoto KS et al, 2009 [38] | The study was conducted between three study groups:  Community Health Worker (CHW) (***CC1***): In order to provide a culturally acceptable intervention consisting of diabetes education, and monitoring services tailored to the needs of patients, the CHW conducted individual education sessions with patients, their family members, and routine follow ups by telephone. The 10-week education sessions were based on ADA standards, but tailored to the needs of the patients. Routine telephone follow ups were conducted to monitor progress, identify problems and assist in problem solving. On an average, 11.3 sessions (per patient) were conducted during the intervention period. The sessions were in addition to standard care.  Case Management (CM) ***(CC2)***: Participants received diabetes related care, education (ADA based) and monitoring from ‘linguistically competent’ & ‘culturally sensitive’ nurses, in addition standard care. The intervention was tailored to meet the patients’ need. Patients met the nurses on a monthly basis or as required at the clinic; the nurses conducted follow up telephone calls when they felt it was required. The nurses also conducted activities, such as: development of a case management treatment plan that incorporated the provider recommended treatment, coordination & referral of community resources, and participation in multidisciplinary case conferences to discuss patient status.  Standard provider care ***(CC3)***: Patients received only standard care by physicians and nurse practitioners  **Impact**: Increased intake in fruit and vegetables (***CC1, CC2***); decreased intake of fatty food, and improved physical activity (***CC1, CC3***). |
| Clarke A, 2009 [39] | Intervention: The intervention consisted of routine educational sessions on information about diabetes and skills for diabetes management. The sessions were given by diabetes nurses as ‘presentations’. The sessions lasted for two hours, with a follow-up session two weeks later on advanced knowledge and skills.  **Impact:** Significant improvement in dietary management behaviour, one month post intervention with the effect being sustained until the six months assessment. |
| Glasgow RE et al, 2009 [40] | The study evaluated the impact of two conditions, in person classes and DVD- delivered DSME.  Classes: Patients attended ‘taking charge of your diabetes’ course, held in two sessions, 2.5–3 h each, conducted by professionals. The curriculum focussed on the ‘AADE7’ key self-care behaviours for diabetes management, namely healthy eating, being active, monitoring, taking medications, problem-solving, healthy coping and reducing risks. Instructors facilitated the group discussion around each topic and encouraged participants to: (1) Self-assess (2) Share information and (3) Set specific goals in each area. Each member attending class was given the diabetes manual.  DVD (as comparison group): Patients received the DVD, titled ‘Diabetes and balance: my personal roadmap’, designed by Kaiser Permanente Colorado diabetes educators that covered the same content areas as the classes. In addition to an introduction and a summary, the material was divided into 7 chapters focusing on the 7 areas of self- care behaviours. Key concepts were illustrated by high-quality graphics and animations, and video segments described the models from different cultures engaging in self-management behaviours.  Similar to the classes group, a goal-setting and problem-solving approach was utilized. Viewers were instructed to pause the DVD and complete an action plan at the end of each segment, and were also advised to complete the DVD in 2 or 3 separate sessions.  **Impact**: Little difference in the impact between the two groups. |
| Kolawole B et al, 2009 [41] | Intervention ***(I)***: Patients participated in structured diabetes self- management and multidisciplinary education program conducted by Diabetes Association of Nigeria (DAN). The sessions were organized in groups. The patients were members of DAN.  Control ***(C)***: Control group consisted of outpatients (with T2D), who were not members of DAN and had not participated in the structured education program conducted by DAN.  **Impact**: Number of patients adherent to medications were significantly higher in intervention group; patients with good knowledge of diabetes and good knowledge of hypoglycaemia were significantly more in the DAN group |
| Mullan RJ et al, 2009 [42] | Intervention ***(I)***: Six medication choice decision aid cards consisting of information on weight change, low blood sugar, frequency of blood sugar testing, daily routine, HbA1c reduction and side effects, for different OHAs were shown to the patients, and they were asked to make a decision about the preferred agent for managing their blood glucose (discussion between clinician and patients to choose the treatment agent). Copies of the cards were provided as pamphlet to the patients to take home.  Control ***(C)***: Discussion about the OHAs was carried out without the treatment aid cards with the clinicians in the ‘usual manner’. Patients were provided a 12- paged general pamphlet on OHA to take home.  **Impact:** The decision aid was acceptable and helpful to the patients and physicians; intervention patients scored significantly higher on knowledge questions pertaining to information; the decision aid was effective in promoting patient involvement in decision making process. |
| Rodin HA et al, 2009 [43] | Intervention: Pharmacy-benefit change that introduced the provision of free generic medications and higher co-payments for brand- name medications.  **Impact**: The significant improvement in adherence seen only with statins; change from brand to generic and cost analysis resulted in inconsistent findings |
| Sacco WP et al, 2009 [44] | Intervention ***(I):*** The intervention consisted of brief, regular, telephone coaching sessions provided. Patients received telephone coaching sessions 1 call/ week for first 3 months and then one bi- weekly for the remaining 3 months. Telephone sessions were guided by a weekly coaching checklist and addressed the following areas of self-care: blood sugar testing, medication management, diet, exercise, foot care, stress management and other relevant issues, for example: eye examination, dental care and vaccinations. Weekly goals were assessed and when goals were not met, the participant was engaged in collaborative problem-solving to overcome the challenges. Effort and positive changes were praised.  Control ***(C)***: Received usual treatment from endocrinologists and completed pre- &post-test measures, approximately 6 months apart.  **Impact**: Adherence to diet, exercise and foot care; depressive symptoms; diabetes symptoms; self- efficacy; healthcare team support; reinforcement for self-care; and awareness of self-care goals improved significantly |
| Thoolen BJ et al, 2009 [45] | Intervention ***(I)***: Intervention consisted of 2 individual & 4 group sessions spread over 12 weeks. The first individual session consisted of discussion on experiences with diabetes followed by group sessions, each of which consisted of a brief discussion on self- care themes, goal-setting and motivating the patients to work towards personally set goals. The process for evaluating their progress was considered in advance. The group sessions were then followed by the 2^nd^ individual session, which evaluated the progress and made plans for the future  Control ***(C)***: Participants were provided a brochure on diabetes self- management  **Impact:** Patients who completed the course performed significantly better on proximal measures, such as intention, self- efficacy and proactive coping, when measured at 3 months and 12 months (not significant compared to baseline; group *time differences were significant) |
| Adepu R, Ari SM, 2010 [46] | Intervention ***(I)***: The intervention group received education regarding disease, medication, diet and lifestyle modification at baseline and at each follow up (at an interval of 30 days for 3 months)  Control ***(C)***: Participants received detailed education only at the final follow- up (after completion of post data collection)  **Impact**: Significant improvement in medication adherence in the intervention group |
| Bogner HR, de Vries HF, 2010 [47] | Intervention ***(I)***: In collaboration with physicians, a care manager offered guideline-based treatment recommendations to patients in order to help them ‘*recognize depression in context of T2D’,* and monitored adherence, clinical status, and provided appropriate follow-up. The key components of the intervention were (1) provision of an individualized program (which recognized patients’ socio- cultural context) to improve medication adherence and (2) integration of T2D treatment with depression management. The intervention consisted of three 30-minute in-person sessions and two 15-minute telephone monitoring contacts over a 4-week period, during which the patients received education about depression and diabetes; the importance of depression control for T2D management was emphasized; issues relating to stigma, symptom identification for depression and T2D, rationale for medication usage, medication management and monitoring were addressed and assistance provided. The diabetes information for participants was tailored to their cultural context.  Control ***(C)***: At baseline, 2, 4, 6, and 12 weeks, participants underwent assessments without the intervention.  **Impact**: Significantly greater number of participants with >80% adherence to oral hypoglycaemic and anti-depressants medication in the intervention group; HbA1c mean and Center for Epidemiologic Studies Depression Scale (CES-D) mean improved significantly in the intervention group. |
| Borges APDS et al, 2010 [48] | Intervention ***(I)***: The patients had standard care and were followed up monthly by a single clinical pharmacist, who collected patient data &monitored them and prepared records which contained patients’ data on admission, laboratory data, and compliance test data conducted on follow- up and the pharmaceutical care intervention plan developed for each patient. In addition to individual patient education conducted depending upon the needs and knowledge about medicines and disease, the pharmacists also performed verbal and written guidance to disease control, compliance to treatment, appropriate nutrition and correct use of medicines. The pharmacist also collaborated with other health professionals, to perform dose adjustment, drug therapy modification, and modification of diet plan and care of the diabetic foot, and planned referrals as required. All the plans, process and outcomes were documented.  Control ***(C)***: Participants only received the standard care provided by the endocrinology unit (appointments every 4 months). The procedures were registered in the patient records and could consist of alterations in the prescribed drugs, requests for laboratory exams, general information about patient health and referrals to specialists. There was no contact between the pharmacist and the participants.  **Impact**: HbA1c improved significantly in the intervention group compared to the control group; improvement was also seen in fasting glycaemia in intervention group post intervention; a significant improvement in the identified drug therapy problems (more than 50% in most cases were solved, except for the instances where drug doses were too high); medication adherence improved post intervention in the intervention group. |
| Castillo A et al, 2010 [49] | Intervention ***(I)***: Community health workers (CHWs) were trained using Diabetes Empowerment Education Program (DEEP) (developed by the University of Illinois’s Midwest Latino Health Research training and policy centre) who in turn delivered the intervention to patients with diabetes. Interventions were delivered to CHWs and patients:  1. Intervention to the CHWs: In order to address the ‘capacity building needs’, CHWs underwent the DEEP consisting of 2 components: (1) Training of Trainers (TOT), and (2) Diabetes Education Program. TOT was a 20- hour workshop to prepare CHWs to implement the educational curriculum, to the people in the community. Diabetes education program consisted of a series of educational programs aimed at increasing disease knowledge, developing self- management skills and facilitating behavioural change. The areas covered were ‘diabetes risk factors, diagnosis, treatment, complications nutrition, physical activity, psychosocial aspects, self-care skills, goal setting and identification of important clinical markers, effective communication with providers, and utilization of community resources’. The curriculum focused on the cycle of knowledge- reflection- action and allowed CHWs to gain understanding of their clinical, social, emotional context. Informed decision making was facilitated. In addition to the DEEP, CHWs also received training on other important issues, for example ‘human subject’s protection’, use of BG and BP monitors, and the DCA 2000+ analyser for the evaluation of HbA1c.  2. Intervention to the patients: The intervention consisted of 2- hour group sessions (every week for 10weeks) led by a team of 2 CHWs (facilitator and assistant). First week, however, was dedicated to registration and collection of consent form and baseline data. Participants then attended 8 weeks of group education sessions, conducted in groups of 10- 15 participants, including family and friends. The sessions were conducted in English or Spanish. The last week (the 10th week) was also dedicated to collecting post data and celebrating graduation. Facilitators maintained regular contact with their participants to motivate attendance.  **Impact**: A significant increase in diabetes knowledge and improvements in physical activity and nutrition practices (except the reduction of high-fat foods);self-care practices such as foot care, self-monitoring of glucose levels, and adherence to medications also improved significantly; the patients showed a significant reduction in HbA1C and systolic blood pressure |
| Cinar FI et al, 2010 [50] | Intervention: The intervention consisted of telephone interviews and follow up calls conducted by nurses. During the first interview, patient information such as, demographics, medication status, diabetes knowledge, adherence to different self-care measures and metabolic control results were recorded. During the same call, an average of 30-minute diabetes education (about the condition and its risk factors, diet, exercise, drug therapy, glycaemic management) was given, along with identification of problems (and appropriate solutions) related to control . Whenever possible, patients’ relatives were included in the training. Patients were called once a week in the first month and once every two weeks in the following months (average call: 8/ patient). During each call, diet, exercise, and medication adherence were questioned and additional problems (if any) were identified and recorded to be followed up in the next call. Problems identified during phone calls, suggested solutions, next interview dates, problems and evaluations to be discussed at the next interview, were recorded.  **Impact**: Number of patients adherent to medication, diet and exercise significantly improved post intervention; metabolic parameters like HbA1c, fasting and Post Prandial BG, Triglyceride and LDL levels, BMI, and Blood Pressure improved significantly. |
| Gonzalez JS et al, 2010 [51] | Intervention ***(I)***: The intervention consisted of 1 visit with a nurse diabetes educator, 2 with dietician and 10-12 sessions of Cognitive Behavioural Therapy for Adherence and Depression (CBT-AD).Session started with a focus on adherence to medical recommendations, followed by other sessions that consisted of informational, problem-solving, and cognitive behavioural steps that targeted a range of self-care behaviours. To help patients change their cognitions about self-care behaviours by eliciting positive reasons for being adherent, patients were encouraged to focus on such thoughts when engaging in such behaviour. Goal setting and moving towards the goal—changing the goal as required, were incorporated. CBT-AD formed the core component.  **Impact**: Improvement in depression scales, adherence to self-care measures like medications, diet, exercise, foot care and glucose monitoring and HbA1c. |
| Tang TS et al, 2010 [52] | Intervention ***(I)***: Intervention consisted of weekly sessions over a period of 24 months (however, the study analysed data collected in the first 6 months only). Discussions were based on patients’ questions, concerns, and priorities. The content of sessions were patient-directed, however, the intervention involved 5 core processes: (1) reflecting on relevant self-management experiences, (2) discussing emotions and feelings,(3) engaging in problem-solving, (4) addressing questions about diabetes and its care, and (5) behavioural goal-setting. Participants were invited to attend sessions as frequently as they required.  **Impact**: Participation in the intervention led to a significant reduction in HbA1C |
| Wolever RQ et al, 2010 [53] | Intervention ***(I)***: Intervention consisted of ‘integrated health (IH) coaching’. Each participant was paired with a ‘coach’ who would deliver the intervention. The participant – coach pair remained the same throughout. ‘Wheel of Health’ was administered during the initial assessment visit, to direct the conversation, to assess success, to explore values, establish priorities and set goals. Within 2 weeks of the baseline visit, first telephone session took place, during which the participants were asked by the coaches, in relation to their diabetes management ‘what was important to them’, ‘how well they were managing their health’, and ‘what they perceived to be the challenges or areas of required support’. Patients were directed towards creating a ‘vision of health’, and long term goals that aligned to the vision were discussed. Based on assessment using the Wheel of Health, areas in which patients felt less successful were identified and patients chose areas that needed coaching. The coaches used the following prompts “how will goals in this area support the bigger picture of your life?” “how will your life be better?” and “how will this enable you to meet your purpose, as you see it, in this world?”. The domains in the Wheel of Health were discussed in the remaining sessions. The vision guided the formation of the goals and the goals were further broken down into small, realistic action steps. Fourteen telephone call sessions (8 weekly calls, 4 biweekly calls, and a final call 1 month later), each lasting for 30 minutes, were conducted after the initial call. The patients were also provided with a binder of educational materials at the initial assessment visit, with a motive to facilitate their learning.  Control ***(C)***: Participants did not receive the IH coaching.  **Impact**: Perceived barriers to medication adherence decreased, while patient activation, perceived social support, and ‘benefit-finding scale’ improved in the intervention group compared to control. Improvements were also observed for self-reported adherence, exercise frequency, stress, and perceived health status in intervention group. Intervention participants with elevated baseline HbA1c (≥7%) had significant reduction in their HbA1c. |
| Zhang Y et al, 2010 [54] | Intervention ***(I)***: The impact of implementation of Medicare Part D program was evaluated, using three study groups: No coverage group: had no drug coverage; $150 cap group: had a drug coverage with a $150 quarterly cap; and,$350 cap group: had a drug coverage with a $350 quarterly cap  However, before implementation of Part D program, $150- cap and $350 cap groups paid an $8 or $20 co-payment, respectively, before their total pharmacy spending reached the quarterly cap, and paid full drug costs after reaching the cap level per quarter.  Comparison ***(CC)***: Throughout the study period, was covered by retiree health benefits, had no deductions and had to pay $10/ $20 per monthly prescription irrespective of their total spending  **Impact**: Medication adherence for anti-diabetics improved; improvements were seen with treatment intensity, likelihood of medication adherence for hypertension and hyperlipidaemia drugs. |
| Gracia- Huidobro D et al, 2011 [55] | Intervention ***(I)***: The intervention incorporated family members in the care of their diabetic relatives. The patients in the intervention clinic participated in 2 interdisciplinary family meetings or home visits where providers, guided by semi-structured interviews, talked about family or other psychosocial factors that could interfere with diabetes control and at the end, made the patients and relatives sign an agreement making a commitment to change. During home visits, families were provided with a recipe book for diabetes and during family meetings, with a framed family picture. Patients also received an individual counselling session (at least one) and one counselling session with their relatives where the importance of family support strategies were discussed. Patients and relatives were encouraged to attend multifamily group sessions, to discuss health behaviours and control strategies. As part of the intervention, the organization of the clinic was changed towards a family-oriented health centre, where all members of the health care team promoted family participation in the care of patients with T2D.  Control ***(C)***: Two clinics were assigned as control clinics, control clinic 1***(C1)*** and control clinic 2 ***(C2)***. Patients in both clinics received usual care as per the guidelines for T2D of the Chilean Ministry of Health.  **Impact**: HbA1c was significantly reduced in the intervention clinic at the second 6 months of intervention time point; there was a significant reduction in depressive symptoms in the intervention clinic compared to ***C1***. |
| Khan MA et al, 2011 [56] | Intervention ***(I)***: Patients viewed a computer multimedia program ‘Living Well with Diabetes’ in the waiting-room setting of a county clinic. The program consisted of: ‘an introduction to diabetes, BG management, oral-medications and insulin, nutrition and physical activity, depression and stress, oral hygiene, and prevention of complications’. Video stories, graphic animations, professional narration, interactive quizzes, and feedback were the strategies included. Testimonials from different subjects were used with culturally-appropriate information for the users.  Control ***(C)***: Patients read an educational brochure while in the waiting room.  **Impact**: Intensification of therapy (oral diabetes medications) was significant in the multimedia group post intervention; After 3 months, a near significant (p=0.006) reduction in HbA1c was observed among intervention participants. |
| Mehuys E et al, 2011 [57] | Intervention ***(I)***: Participants received a ‘protocol- defined intervention’, at the start and at each prescription refill visit, consisting of: (i) education about type 2 diabetes and its complications (ii) education about the correct use of OHAs (timing in relation to food) (iii) facilitation of medication adherence (iv) healthy lifestyle education (diet, physical exercise and smoking cessation) and (v) reminders about annual eye and foot examinations.  Control ***(C)***: Participants received usual pharmacist care.  **Impact**: Significantly more pharmacotherapeutic changes; significant improvements in HbA1c; in knowledge about diabetes, and in domains of specific diet, physical exercise and foot care in the intervention group. |
| Mitchell B et al, 2011 [58] | Intervention: The intervention consisted of a Diabetes Medication Assistance Service (DMAS).Patients received a MediSenseOptiumXceed^TM^ blood glucose (BG) meter on their first visit, were instructed on its use, and asked to take measurements at least once daily (preferably at different times). During the next 4 visits, the pharmacists downloaded each patient’s BG reading using Precision Link Direct Device^TM^, generated printouts and charts of their BG results, which were used to conduct discussions between the patient and pharmacist in areas of glycaemic control. ‘Self -management support interventions (SMSI)’ were then delivered based on pharmacists’ assessment of patients’ condition, the BG readings and patients’ concerns. Goals to be achieved by next visit were discussed, negotiated and documented. An SMSI was defined as “*any action taken by a pharmacist during the consultation to address issues relating to self-monitoring of BG, medication adherence, medication problems or lifestyle such as exercise, diet, and foot care.*”  **Impact**: Regimen screen and belief screen scores of the Brief Medication Questionnaire (BMQ) were significantly better; significant reduction in mean BG level; more than 90% of patients reported improvements in their knowledge about diabetes self-management |
| Piette JD et al, 2011 [59] | Intervention***(I)***: The intervention consisted of a telephone delivered cognitive behavioural therapy (CBT) program, and included an initial intensive phase of 12 weekly sessions followed by 9 monthly booster sessions. Initially the intervention focused on patients’ depressive symptoms. After 5 sessions, pedometer based walking program, was introduced in addition to the links between depression, physical activity and diabetes outcomes. Manual logs were provided for patients’ use to do ‘CBT homework exercises’ and to track their progress toward step-count goals. An introductory letter was sent to the patient’s provider after recruitment. The providers received summary reports on Patient Health Questionnaire (PHQ) scores every 3 months or more frequently if needed, and were notified when an urgent attention was required for example of suicidal ideation, and discontinuing anti- depressant medication on their own.  Control***(C)***: The participants received a copy of the ‘Feeling Good Handbook’ (a self-help book based on CBT for depression), educational materials about depression, walking and diabetes, and a list of local resources for depression. With the patient’s consent, their primary care physician was informed about their depression scores.  **Impact**: Blood pressure measures, step counts and Beck Depression Inventory scores improved significantly post intervention in the intervention group. |
| Ramanath KV, Santhosh YL, 2011 [60] | Intervention ***(I)***: Intervention consisted of the supply of educational materials, patient information leaflet and formal counselling to the participants by a clinical pharmacist  Control ***(C)***: The educational material and counselling was given to control group only at the end of the study.  **Impact**: Significant improvement in adherence with respect to control; reduction in blood glucose level in last follow up in intervention group; significant improvement in knowledge, attitude and practice behaviour in the intervention group |
| Shetty AS et al, 2011 [61] | Intervention ***(I)***: Patients were delivered SMS once every 3 days to remind them to strictly follow the dietary modification regimen, physical activity and medication schedules  Control ***(C)***: Patients received standard care (that consisted of appropriate medications prescribed and advice on diet modification and physical activity)  **Impact**: HbA1c, plasma lipids and post prandial blood glucose improved significantly in the intervention group |
| Smith SM et al, 2011 [62] | Intervention ***(I)***: Practices allocated to intervention group, introduced a ‘structured diabetes care system’ (involving regular recall of patients every 3 to 6 months with an annual audit of risk factors). Three individuals were recruited as ‘peer supporters’. The peers were trained regarding the basics of T2D and issues relating to working with groups and confidentiality, and advised that their role in the intervention would be to provide social support, rather than health care or education. The patients attended 9 peer support meetings over the 2 year period, facilitated by peer supporters. Each meeting had a suggested theme and a small structured component and at the end of the session, frequently asked questions were collected from all groups and sent back for the next session. In addition, the peer supporters were in contact with the participants via telephone calls and letters  Control ***(C)***: Practices in the control group also introduced the ‘structured diabetes care system’, but peer supporters and their intervention was not present.  **Impact**: Nothing significant |
| Wakefield BJ et al, 2011 [63] | Intervention ***(I)***: The intervention used a home tele-health device (device used a standard phone line to enable data transmission between the patient’s home and the study centre), where patients entered BP and BG measurements, and answered standard questions based on which group they were assigned to. Patients received an automated response based on their data and answer to the questions (‘correct responses were reinforced and incorrect responses were reviewed and explained’). The device also allowed individualized messages to be transmitted to subjects. The data entered by patients were automatically downloaded each night enabling review the next day, and quick intervention when required. The patients in the intervention were divided into two groups: High Intensity ***(I1)*** and Low intensity ***(I2)*** groups(see below). Each day, the responses were reviewed and the follow- up delivered (that is, additional health information, increased monitoring, compliance strategies, problem resolution facilitation, or contact with the subject’s physician) if required. Follow up was conducted by telephone, mailed letters or using the tele-health device to send a message. The intervention was carried out for 6 months.  ***I1***: For the participants in the ‘High-intensity group’, the study team, developed a ‘branching disease management algorithm’ based on DM and HTN guidelines from the VA, American Diabetes Association, and the American Heart Association, which was then programed into the device. The algorithm focused on relevant issues, for example, diet, exercise, smoking cessation, foot care, advice for sick days, medications, weight management, and preventive care. The subjects received the standard prompts each day, and a rotation of questions and educational content (on a scheduled time) basis based on the algorithm.  ***I2***: The participants in ‘Low-intensity group’, responded to a small subset of questions from the larger set used with the high-intensity group. Each day, subjects were asked ‘Have you taken all your medications as prescribed?’ and one additional question focused on diet, exercise, foot care, or medication side effects. Branching algorithm was not used; rather a yes/no or multiple choice responses were sought.  Control ***(C)***: Usual care subjects scheduled follow-up appointments with the primary care clinic in the usual manner.  **Impact**: Improved HbA1c during the 6- months, but after the intervention was withdrawn at 6 months, the intervention groups were comparable to control groups; the high intensity patients had a significant decrease in systolic blood pressure compared with the other groups at 6 months |
| Walker EA et al, 2011 [64] | The study compared a telephone intervention with a print intervention.  Telephone intervention ***(CC1)***: The participants received up to 10 telephone calls from their health educators at 4- to 6- week intervals. Although the calls were tailored to individual participant’s reported needs, the focus was on diabetes medication adherence and lifestyle changes through healthy eating and physical activity. Problem solving, goal setting, communication skills, and preplanning for medical visits formed important elements. Participants were encouraged to choose topics for each call, despite the call being guided by a manual. Selected ‘high quality self-management materials’ were mailed to each participant, and were prompted to use them. .  Print intervention ***(CC2)***: Participants received ‘selected high-quality self-management materials’ by mail  **Impact**: Telephone intervention had a significant impact on medication adherence among the patients not using insulin; HbA1c was significantly improved in the telephone intervention group |
| Barron JJ et al, 2012 [65] | The manuscript discusses two studies, one that evaluated the impact of co-payment reduction (Study 1, prior approval (PA)) and the other that evaluated the impact of waived co-payments and a mandatory telephone diabetes education support (TDES).  **Study 1 (PA):**  Intervention ***(I)***: Participants had a 50% reduction in co-payments on a prior approval (PA) basis  Control ***(C)***: Participants had no co-payment reduction  **Impact**: Nothing significant  **Study 2 (TDES)**:  Intervention ***(I)***: Participants took part in a Value –Based Insurance Design (VBID) program that provided waived co-payments for participation in a mandatory TDES initiative  Control***(C)***: Participants did not have co-payment reductions or diabetes education support  **Impact**: Significantly better comprehensive diabetes care, as measured by a significantly higher percentage of patients receiving HbA1C tests, LDL- tests, kidney tests and eye examinations |
| Bogner HR et al, 2012 [66] | Intervention ***(I)***: Integrated care manager in collaboration with physicians offered education and guideline-based treatment recommendations to patients, and monitored adherence and clinical status. They worked individually with patients to address the factors involved in adherence. Different factors were addressed via three, 30-minute in-person sessions (at baseline, 6 weeks, and 12 weeks) and two, 15-minute telephone-monitoring contacts over a 3-month period. Through these sessions, education about depression and T2D, was provided emphasizing the importance of controlling depression to manage diabetes, and helping patients identify and understand target symptoms, rationale for antidepressant and OHA use, methods of assessment for side-effects and provided assistance in their management; along with assessment for progress; assistance with referrals; and monitoring and response to life-threatening symptoms. The intervention was in addition to existing primary care treatment.  Control ***(C)***: At baseline, 6, and 12 weeks, participants underwent the same assessments as participants in the intervention.  **Impact**: Improvement in medication adherence; patients in intervention group had a significantly improved mean change in HbA1c levels and Patient Health Questionnaire (PHQ-9)scores; intervention patients were more likely to achieve targeted HbA1c (<7%) and to achieve remission of depression compared with patients in the usual care. |
| Brennan TA et al, 2012 [67] | Intervention ***(I)***: Patients received a follow- up call in response to late refill of their anti- diabetic medications, with an offer for a refill and a discussion on adherence. Patients starting a new medication were also counselled at the time of their first fill of the medication. For those patients in the retail setting who were diabetic but not prescribed with statins, angiotensin-converting enzyme inhibitors, or angiotensin receptor blockers, the retail pharmacist counselled the patient about the need for such medications. The pharmacist also offered to contact the patient’s physician to discuss the medication use. If the patient consented, the prescriber was sent a fax and asked if he/ she wished to start the medication(s). The patient was informed about the prescriber’s decision. Similar effort was made by the mail order pharmacist, based on phone outreach to the patient and fax to the doctor.  Control ***(C)***: Control group consisted of people with diabetes, based on a ‘roughly 5:1 match’ with program participants.  **Impact**: Medication adherence rates were improved; the estimated improvement in the initiation rates of concomitant therapy, Angiotensin Converting Enzyme Inhibitors and Angiotensin Receptor Blockers during the intervention was significant in both the groups. |
| Choi SE, Rush EB, 2012 [68] | Intervention: The intervention consisted of 2 sessions of diabetes self- management education. The sessions lasted for 1.5 and 2.5 hours, respectively. 2^nd^session occurred 2 weeks after the 1^st^ session. The education was based on content considered essential by the ADA and the National Diabetes Education program and the instruction included pathophysiology of diabetes, complications, treatment modalities, medication, diet, exercise, and self-management strategies, along with self-monitoring of BG and its interpretation. The intervention employed the native language, integrated cultural dietary preferences, encouraged family participation and support, and held open discussions of cultural beliefs and treatment practices for diabetes. Traditional food recipes, nutritional label reading, carbohydrate and calorie counts were taken into consideration, along with the provision of counselling for dietary change by modification of ethnic recipes, demonstration of healthy food choices, and cooking tips. Intervention also included assistance in developing individualized plans for preventing and treating diabetic complications and visual counselling for foot and skin care.  **Impact**: Significant decrease in HbA1c, increase in HDL, decrease in waist circumference; number of reported feet checks per week increased significantly across the 3 assessments. |
| Farmer A et al, 2012 [69] | Intervention ***(I)***: Patients’ usual prescription for OHAs was dispensed in the electronic medication device, and the use of the dispenser was explained to the participant. Participants were then asked to come for the follow- up and intervention visit after eight weeks.  At the intervention visit, participants took part in a consultation with a clinical nurse, during which the nurse delivered the intervention (approximately 30 minutes). The intervention consisted of two components 'motivational component' and 'action planning component'. In the first component, the nurse explored patient's perception about the benefits and harms of taking medication, factors that promoted and hindered daily medication taking behaviour along with the views of other people who were important to the patient. Based on the feedback the nurses reinforced positive beliefs through tailored information using verbal and non- verbal approaches. For the negative beliefs, problem solving was facilitated. In the action planning component, the patients were asked to generate and jot down the exact circumstances in which they would take their medication (using an “if-then” formulation to elicit where, when and how this would occur). The intervention was carried out in weeks 9- 20 of 20 week study.  Control***(C)***: Participants received their anti- diabetic medications in standard blister packs; none of the techniques used for intervention were applied.  **Impact**: Mean percentage of adherent days was significantly better in the intervention group. |
| Kroese FM et al, 2012 [70] | Intervention: The study added booster sessions to the *'Beyond Good Intentions Program*' , which had been evaluated and proven successful in previous research, and studied the impact of booster sessions. The Beyond Good Intentions Program consisted of sessions (1 individual and 4 group) delivered over a 6 week period that taught participants to: *set small, concrete goals*; *recognize conditions for and barriers to goal achievement*; *come up with problem solving strategies in specific challenging situations*; *formulate action plans, and evaluate their progress.* This program was then followed by 'booster phase'. The booster sessions consisted of 3 group sessions at 1, 3 &6 months after the end of the initial phase. After the initial phase the patients were randomly divided into two groups, referred to as 'booster conditions': the **'how'** and the **'why'** condition. The content of the ‘how’-condition was similar to the initial phase, the Beyond good intention program. Participants were instructed to convert, stepwise, their self-management goals to concrete sub-goals (e.g. ‘*How are you going to get more exercise?*’). In the ‘why’-groups, the participants were asked to consider their self-management goals in alignment to their visions, *the higher-order overarching goals* (e.g. ‘*Why are you going to get more exercise?’*).  **Impact**: No additional improvement was seen with the addition of booster sessions |
| Mellitus Janice C-M et al, 2012 [71] | Intervention: Termed as Diabetes Self- Management Education (DSME)intervention, patients attended sessions (which focused on 7 areas of T2D self-care, recommended by ADA and AADE : (1) healthy eating, (2) being active, (3) monitoring blood glucose, (4) taking medications, (5) problem solving,(6) reducing risks, and (7) healthy coping;)held once a week for 2 hours over 6 weeks. Along with culturally targeted written materials and videotapes, presentations were provided, where both educational and behavioural strategies, were incorporated. After the sessions were completed, participants were asked to practice self-management behaviours independently for 6 additional weeks.  **Impact**: Significant increase in medication adherence, healthy eating and foot care adherence; a clinically significant effect on systolic blood pressure, blood lipids, physical activity, and waist circumference. |
| Odegard PS, Christensen DB, 2012 [72] | Intervention ***(I)***: Patients were contacted by telephone by their pharmacist following a missed refill date. The phone call was conducted using standard process based on “4 A’s” (ask, advise, assist, and arrange) model. During the call, pharmacists asked if patients had exhausted their medication supply, inquired about the reason for the late refill, identified and addressed adherence challenges, provided tailored diabetes adherence education and encouragement, and developed a self-management action plan. A scheduled follow- up call was planned between 1 week and 1 month after the intervention, to assess needs, confirm problem resolution, and provide self-management encouragement. Pharmacists also reinforced adherence and the intervention messages, if requested by the patients, at the time of in-person medication refills.  Control ***(C)***: No intervention was provided.  **Impact**: MPR was significantly improved in the intervention group: improvement in MPR was greater in patients starting with low MPR (<0.8). |
| Ramanath KV et al, 2012 [73] | Intervention ***(I)***: Patients received counselling on different aspects relating to the disease, drugs and their management, and were provided with disease and drug patient information leaflets at baseline. .  Control ***(C)***: No counselling provided to the control group. They were only advised to come for the follow ups.  Patients in both groups were provided with diary cards as a medication adherence reminder and advised to come for the follow up.  **Impact**: Significant improvement in: medication adherence; overall quality of life, the mental component, role- emotional, social functioning, bodily pain of SF- 12v2 QoL questionnaire; and Fasting Blood Sugar and Post Prandial Blood Sugar was significantly improved in the 2^nd^ follow up |
| Vervloet M et al, 2012 [74] | Participants in both groups received their oral anti-diabetics in the Real Time Medication Monitoring (RTMM) medication dispenser and had their medication use registered in real time for 6 months. The RTMM system consisted of an electronic dispenser, which sent a brief message to a central server each time it was opened. This message received was the information about the date and time of the opening of the dispenser and its identification number. During recruitment in the pharmacy, patients were informed about the study by the pharmacy staff, provided with the dispenser with a brief user instruction, and were asked to choose one, two or three (according to their prescribed number of daily doses)time periods within which they would take their medication. The time periods chosen were communicated to the supplier of RTMM, to program the software accordingly.  Intervention ***(I)***: Participants received a SMS reminder if they had not opened their medication dispenser within the chosen time period, at the end of the time period. The SMS reminder read as follows: “Have you taken your medication yet? Please take your medication as prescribed by your health care provider.” Only one reminder for each dose was sent.  Control ***(C)***: Participants did not receive the SMS reminders.  **Impact**: Significantly more patients in the SMS group than control group reported higher awareness of their medication use; reminded patients took the medication more regularly than those who were not reminded. |
| Zolfaghari M et al, 2012 [75] | The study compared an SMS intervention with a telephone intervention.  Participants in both the groups received 3 days of diabetes self- care education at the beginning.  SMS group ***(CC1)***: Participants received 6 SMS per week for 3 months (72 messages/ participant in total) that consisted of information and optimal recommendations about diet, exercise and diabetic medication taking, frequent self- monitoring of blood glucose levels and stress management.  Telephone group ***(CC2)***: Participants were counselled by telephone on the nature of disease, risk factors, BG recording and maintenance, and reinforcement of diet, exercise and medications taking. Telephone calls were conducted twice a week for the 1^st^ month and then weekly for the 2^nd^& 3^rd^ month. Patients were also asked about the problems they were facing &were advised accordingly, in addition to the advice on stress management.  **Impact**: Significant changes were not seen between groups. However, there were significant differences from pre to post test in both groups for HbA1c, adherence to diet, physical exercise and medication. |
